# Nuclear RNA catabolism controls endogenous retroviruses, gene expression asymmetry, and dedifferentiation

**DOI:** 10.1016/j.molcel.2023.10.036

**Published:** 2023-11-22

**Authors:** Denis Torre, Yesai S. Fstkchyan, Jessica Sook Yuin Ho, Youngseo Cheon, Roosheel S. Patel, Emma J. Degrace, Slim Mzoughi, Megan Schwarz, Kevin Mohammed, Ji-Seon Seo, Raquel Romero-Bueno, Deniz Demircioglu, Dan Hasson, Weijing Tang, Sameehan U. Mahajani, Laura Campisi, Simin Zheng, Won-Suk Song, Ying-chih Wang, Hardik Shah, Nancy Francoeur, Juan Soto, Zelda Salfati, Matthew T. Weirauch, Peter Warburton, Kristin Beaumont, Melissa L. Smith, Lubbertus Mulder, S. Armando Villalta, Kai Kessenbrock, Cholsoon Jang, Daeyoup Lee, Silvia De Rubeis, Inma Cobos, Oliver Tam, Molly Gale Hammell, Marcus Seldin, Yongsheng Shi, Uttiya Basu, Vittorio Sebastiano, Minji Byun, Robert Sebra, Brad R. Rosenberg, Chris Benner, Ernesto Guccione, Ivan Marazzi

**Affiliations:** 1 Department of Microbiology, Icahn School of Medicine at Mount Sinai, New York, NY 10029, USA; 2 Center for OncoGenomics and Innovative Therapeutics (COGIT), Icahn School of Medicine at Mount Sinai, New York, NY 10029, USA; 3 Department of Oncological Sciences, Icahn School of Medicine at Mount Sinai, New York, NY 10029, USA; 4 Programme in Emerging Infectious Diseases, Duke-NUS Medical School, Singapore 169857, Singapore; 5 Department of Biological Sciences, Korea Advanced Institute of Science and Technology, Daejeon 34141, South Korea; 6 Department of Biological Chemistry, University of California Irvine, Irvine, CA 92697, USA; 7 Center for Epigenetics and Metabolism, University of California Irvine, Irvine, CA 92697, USA; 8 Bioinformatics for Next Generation Sequencing (BiNGS) Shared Resource Facility, Icahn School of Medicine at Mount Sinai, New York, NY 10029, USA; 9 Department of Pathology, Stanford University School of Medicine, Stanford, CA 94305, USA; 10 Department of Genetics and Genomic Sciences, Icahn School of Medicine at Mount Sinai, New York, NY 10029, USA; 11 Department of Pediatrics, University of Cincinnati College of Medicine, Cincinnati, OH 45229, USA; 12 Department of Biochemistry and Molecular Genetics, University of Louisville School of Medicine, Louisville, KY 40202, USA; 13 Department of Physiology and Biophysics, University of California Irvine, Irvine, CA 92697, USA; 14 Seaver Autism Center for Research and Treatment, Department of Psychiatry, The Mindich Child Health and Development Institute, Friedman Brain Institute, Icahn School of Medicine at Mount Sinai, New York, NY 10029, USA; 15 Cold Spring Harbor Laboratory, Cold Spring Harbor, NY 11724, USA; 16 Department of Microbiology and Molecular Genetics, School of Medicine, University of California Irvine, Irvine, CA 92697, USA; 17 Department of Microbiology & Immunology, Columbia University Medical Center, New York, NY 10032, USA; 18 Institute for Stem Cell Biology and Regenerative Medicine and the Department of Obstetrics and Gynecology, Stanford University School of Medicine, Stanford, CA 94305, USA; 19 Department of Medicine, Icahn School of Medicine at Mount Sinai, New York, NY 10029, USA; 20 Department of Medicine, University of California, San Diego, San Diego, CA 92093, USA; 21 Department of Pharmacological Sciences and Mount Sinai Center for Therapeutics Discovery, Icahn School of Medicine at Mount Sinai, New York, NY 10029, USA; 22 Black Family Stem Cell Institute, Icahn School of Medicine at Mount Sinai, New York, NY 10029, USA; 23 Global Health and Emerging Pathogens Institute, Icahn School of Medicine at Mount Sinai, New York, NY 10029, USA; 24 These authors contributed equally; 25 These authors contributed equally; 26 Lead contact

## Abstract

Endogenous retroviruses (ERVs) are remnants of ancient parasitic infections and comprise sizable portions of most genomes. Although epigenetic mechanisms silence most ERVs by generating a repressive environment that prevents their expression (heterochromatin), little is known about mechanisms silencing ERVs residing in open regions of the genome (euchromatin). This is particularly important during embryonic development, where induction and repression of distinct classes of ERVs occur in short temporal windows. Here, we demonstrate that transcription-associated RNA degradation by the nuclear RNA exosome and Integrator is a regulatory mechanism that controls the productive transcription of most genes and many ERVs involved in preimplantation development. Disrupting nuclear RNA catabolism promotes dedifferentiation to a totipotent-like state characterized by defects in RNAPII elongation and decreased expression of long genes (gene-length asymmetry). Our results indicate that RNA catabolism is a core regulatory module of gene networks that safeguards RNAPII activity, ERV expression, cell identity, and developmental potency.

## In brief

Torre et al. report a functional role of the RNA exosome and Integrator in regulating cell identity, ERV expression, and RNAPII elongation in embryonic stem cells. Disrupting either of these complexes promotes a totipotent-like 2CLC state, downregulating pluripotency genes, reducing transcriptional elongation, and primarily affecting long, multi-exonic genes.

## Graphical Abstract

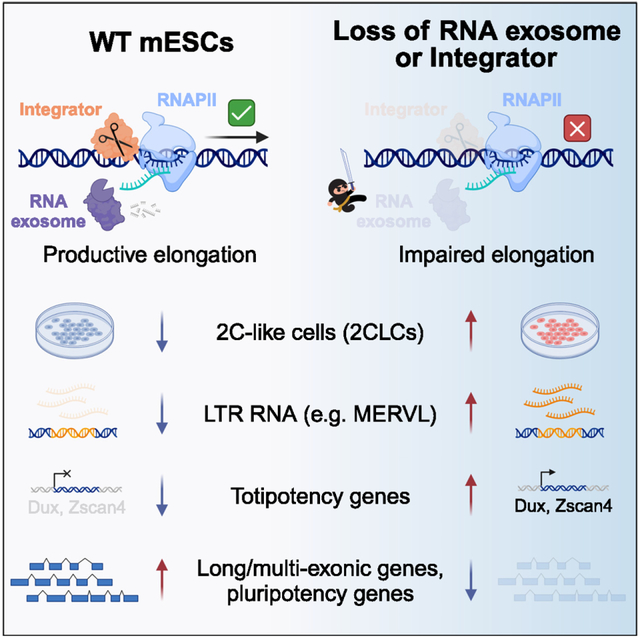

## INTRODUCTION

Epigenetic modifications of chromatin (i.e., DNA methylation and histone post-translational modifications) contribute to segregating chromosomal regions that are accessible and permissive to transcription from those that are not.^1^ This partitioning allows cells to repress the transcription of unwanted coding and non-coding RNA (ncRNA) while enabling the constitutive expression of housekeeping genes and the inducible expression of genes in response to signaling cues.^2^ Many transcription-coupled (e.g., splicing and 3′ end maturation) and post-transcriptional mechanisms (e.g., RNA export) control the outcome of gene expression. Although these mechanisms contribute to the “making” of RNA, catabolic mechanisms regulate RNA levels through degradation. RNA degradation is catalyzed by dedicated enzymes (ribonucleases) that function in both nuclear and cytosolic cell compartments.^3,4^ How RNA degradation sculpts gene expression is not fully understood. This relationship may be of particular importance when considering the degradation of nuclear RNAs, which occurs constitutively on ncRNAs transcribed by active regulatory regions in the genome, such as promoters and enhancers.^5–10^ It is unlikely that RNA synthesis followed immediately by RNA destruction is simply a futile process, as suggested by the pervasiveness of this mechanism.^11,12^ However, how this process impacts the chromatin landscape and the expression of genes controlling cellular states is still incompletely understood.

The mammalian genome contains thousands of endogenous retroviruses (ERVs), which are a subclass of transposable elements (TEs) whose expression is tightly regulated. ERVs play an important role in both embryonic development and different types of diseases.^13–15^ Although heterochromatic ERVs are silenced by a variety of epigenetic mechanisms,^16–21^ we hypothesize that ERVs that reside in euchromatin are regulated at the level of RNA degradation. Under this premise, defects in the RNA degradation machinery could lead to euchromatic ERV de-silencing and functionalization of ERVs, with a potential outcome of affecting cell state identity.^22,23^

To test our hypothesis, we utilized mouse embryonic stem cells (mESCs) and epiblast-like cells (EpiLCs). These model systems enable the study of developmental cell-fate transitions that occur during embryogenesis, which are accompanied by the expression of distinct ERV families.^13,22,24–27^ Our results indicate a key role for RNA degradation in the quality control of RNA polymerase II (RNAPII) transcription, euchromatic ERV silencing, and the regulation of the developmental clock of cell potency in preimplantation development.

## RESULTS

### Loss of Exosc3 upregulates LTR-containing TEs in mESCs

**Figure 1.**
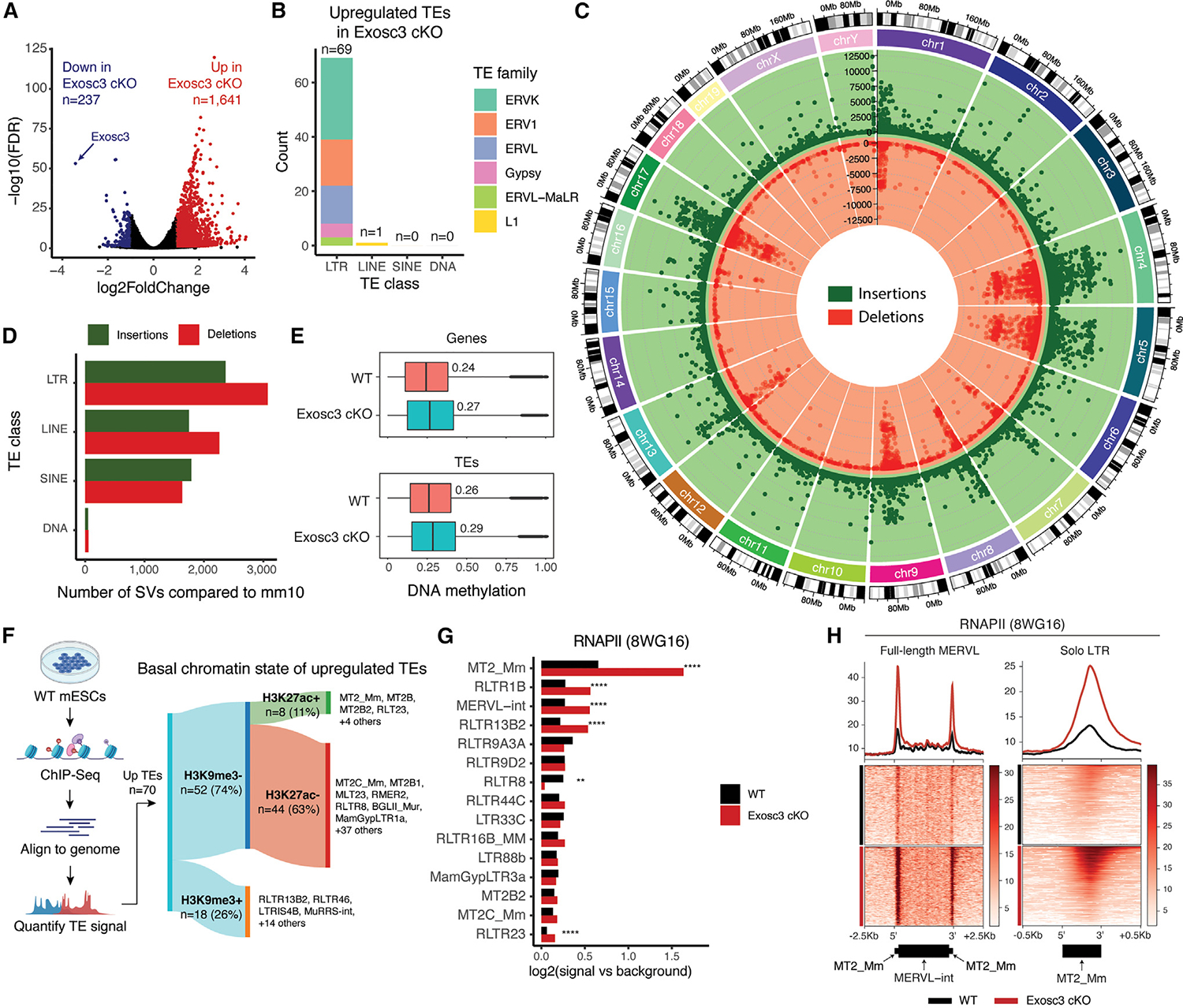
Loss of Exosc3 upregulates LTR-containing TEs in mESCs (A) Volcano plot of differentially expressed genes and TEs between WT and *Exosc3* cKO mESCs. Genes that are significantly upregulated and downregulated in *Exosc3* cKO mESCs are shown in red and blue, respectively (|log_2_-fold change| > 1, p < 0.05, Benjamini-Hochberg correction, calculated using DESeq2). Total number of significantly upregulated and downregulated genes is indicated. (B) Bar plot displaying the number of TEs significantly upregulated in *Exosc3* cKO mESCs. (C) Circos plot of PacBio *de novo* sequenced genome of *Exosc3* COIN mESCs compared with mm10. Green represents sequences within the new assembly, but not in mm10 (insertions). Red represents sequences not found in the new assembly but found in mm10 (deletions). The y axis indicates the size of the structural variants in base pairs (positive values for insertions, negative values for deletions). (D) Number of novel inserted (green) and deleted (red) TE copies in the *de novo*-sequenced genome compared with mm10. (E) Box plots displaying DNA methylation at genes and TEs in WT and *Exosc3* cKO mESCs. (F) Characterization of the basal chromatin state of TEs upregulated in *Exosc3* cKO using ChIP-seq data. (G) Bar plots displaying RNAPII levels at TEs in WT and *Exosc3* cKO mESCs (log_2_-fold change of ChIP-seq signal vs. background, 8WG16 antibody, top 15 TEs displayed). p-values were calculated using an unpaired, two-sided Wilcoxon rank-sum test and adjusted using the Benjamini-Hochberg method (**p < 0.01, ****p < 0.0001). (H) Enrichment of RNAPII ChIP-seq at full-length MERVL elements (MERVL-int flanked by two MT2_Mm LTRs) and solo LTRs (MT2_Mm not located in proximity offull-length MERVLs) in WT and *Exosc3* cKO mESCs.

To understand the contribution of RNA degradation in shaping the pluripotent transcriptome, we performed short-read Illumina RNA-sequencing experiments and analyzed both protein-coding genes (PCGs) and ncRNA expression levels in mESCs comparing wild type (WT) and counterparts that have undergone a conditional inversion (COIN) of *Exosc3*, an essential subunit of the RNA exosome complex.^6^ We henceforth refer to these cells as WT and conditional *Exosc3* knockout (*Exosc3* cKO), respectively, throughout the manuscript. Consistent with recent reports in which RNA exosome subunits were downregulated by short interfering RNA (siRNA) or short hairpin RNA (shRNA) knockdown (KD),^28–30^ we detected a relative increase in upregulated RNAs (n = 1,641) compared with downregulated (n = 237) RNAs in *Exosc3* cKO compared with WT, most of which are non-coding RNAs and TEs (Figure 1A; Table S1). Among these, we detected significant upregulation of 70 TE classes in *Exiosc3* cKO, compared with WT (Figure 1B). Of the 70 upregulated TE classes in *Exosc3* cKO, 69 were long terminal repeat (LTR) retrotransposons, which account for 10% of the genome. These elements are often referred to as ERVs, as they are extant retroviruses that were integrated into the genome on a deep evolutionary timescale. Among retrotransposons that do not contain LTRs, which account for roughly 30% of the genome, we detect an increase in the level of only one class of LINE1 element (Figure 1B). The 69 upregulated ERV classes in *Exosc3* cKO comprise a total of 68,111 single elements.

Since RNA exosome activity has been linked to heterochromatic silencing of non-LTR retrotransposons (i.e., LINE1) via human silencing hub (HUSH) complex and H3K9me3,^31^ we sought to elucidate whether the upregulated LTR retrotransposons in Exosc3 cKO are found in heterochromatic or euchromatic regions and if their elevated expression correlates with alterations to the local chromatin environment. To avoid potential confounding effects of poorly annotated regions in the mouse reference genome, we first sequenced the genome of our WT ESCs *de novo* using whole genome SMRTseq at ~60-fold coverage. We identified approximately 43,000 structural variants (SVs) of over 20 bp, including 29,603 novel insertions and 13,284 novel deletions across all chromosomes specific to the WT COIN mouse^7^ from which our *Exosc3* cKO cells are derived (Figures 1C and S1A).^6^ We then scanned the nucleotide sequences of these variants using RepeatMasker, revealing that LTRs are the TE class with the largest number of SVs, when compared with mm10 (Figures 1D and S1B–S1D).

We then performed DNA methylation analysis at single nucleotide resolution, as this epigenetic modification is often linked to heterochromatic silencing of TEs in somatic cells. Consistent with the fact that mESCs are hypomethylated, we detected a very low and comparable level of DNA methylation across the genome of both WT and *Exosc3* cKO cells, suggesting that the increase in LTR-containing TE expression in *Exosc3* cKO is not attributable to a loss of DNA methylation (Figure 1E).

We then used chromatin immunoprecipitation sequencing (ChIP-seq) in WT and *Exosc3* cKO to assess changes in the chromatin state of TEs. We found that among the upregulated TEs in *Exosc3* cKO, 18 are significantly enriched for H3K9me3 at basal (WT) state (Figure 1F). In addition, several TEs in this class, including RLTR13B2 and others, also display significant levels of MPP8 (Figure S1E), suggesting that their silencing is controlled by H3K9me3 and HUSH.^31^ By contrast, the remaining 52 classes of upregulated LTR-containing TEs lack H3K9me3 at the basal state, suggesting that their silencing is not directly controlled by HUSH. Further stratification revealed a subset of LTRs containing significant levels of H3K27ac at the basal state, including MT2_Mm, the LTR of ERV class III murine endogenous retrovirus-L (MERVL) elements (Figure 1F), where H3K27ac deposition further increases in Exosc3 cKO (Figure S1F).

Consistently, MT2_Mm has the highest level of RNAPII in WT, with this value further increasing in *Exosc3* cKO (Figures 1G and S1G). We additionally confirmed these data by mapping RNAPII and H3K27ac across the genome at each full-length MERVL element, comprised of MERVL internal regions (MERVL-int) with MT2_Mm (LTR) at both ends and solo MT2_Mm (Figures 1H and S1H). This analysis revealed that the enrichment primarily occurs at internal regions of solo MT2_Mm and the 5′ and 3′ ends of MERVL-int. H3K27ac and RNAPII were found at MERVL in both WT and *Exosc3* cKO, and their levels were increased significantly in *Exosc3* cKO (Figures 1H and S1F–S1H). ATAC-seq analysis of WT and *Exosc3* cKO confirmed these results (Figure S1I), as did the reanalysis of public datasets of cap analysis of gene expression (CAGE) and precision run-on sequencing (PRO-seq)^32^ (Figures S1J and S1K). However, some TEs did not display an increase of RNAPII deposition in *Exosc3* cKO despite the significantly upregulated expression (Figures S1L and S1M).

In sum, most LTR-containing TEs that are upregulated upon RNA exosome depletion are in a genomic configuration resembling euchromatin in WT mESCs. Their upregulation is thus achieved through increased RNA stability and/or transcription activation.

### Loss of Exosc3 increases the developmental potential of mESCs

**Figure 2.**
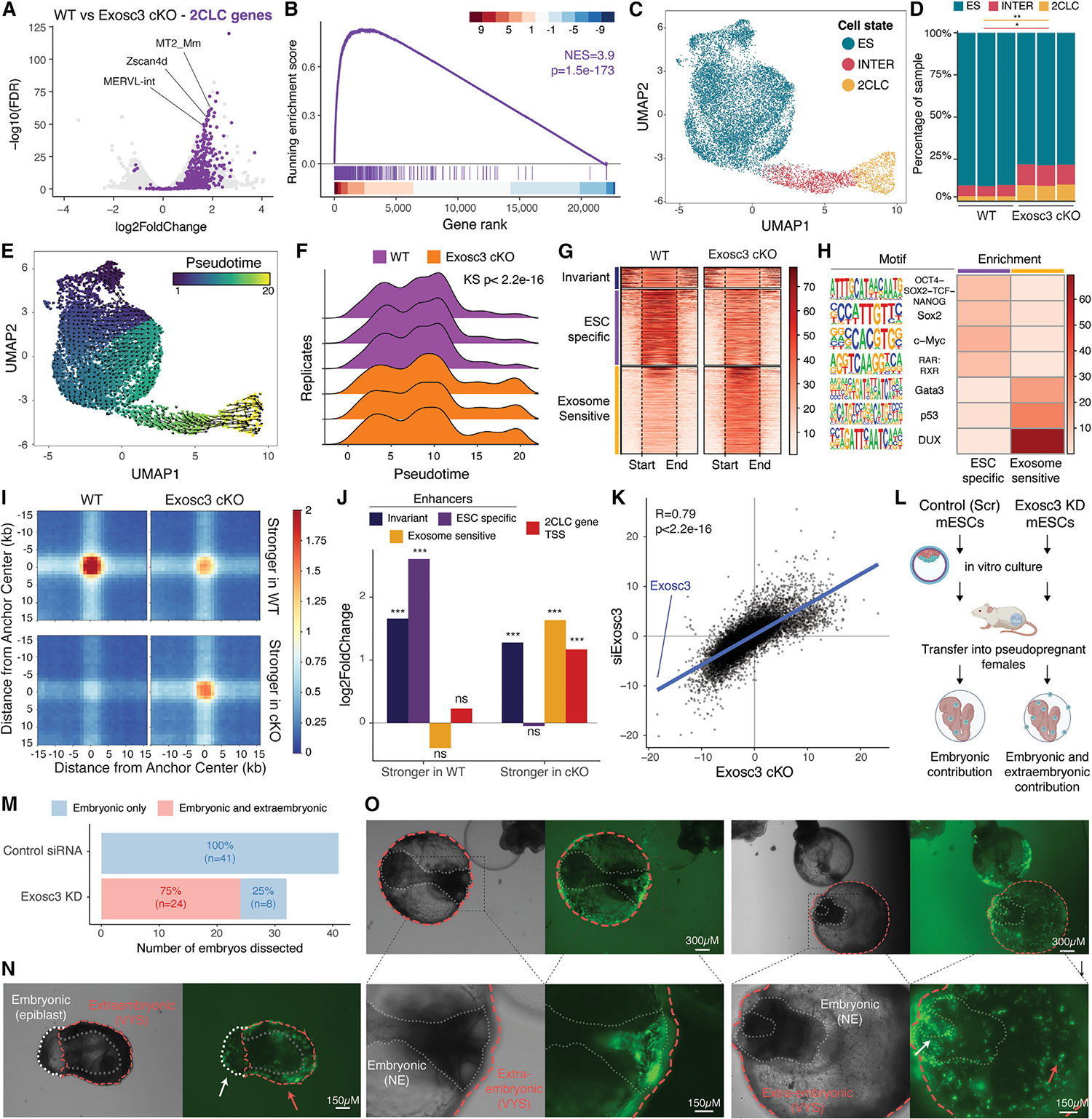
Reduced *Exosc3* levels in mESCs increase their developmental potential (A) Volcano plot of differentially expressed genes and TEs between WT and *Exosc3* cKO mESCs. 2CLC-specific genes and TEs, representing markers of 2CLCs, are highlighted in purple (defined from Eckersley-Maslin et al.^36^). Other genes and TEs are marked in gray. (B) Gene set enrichment analysis (GSEA) of 2CLC genes/TEs in *Exosc3* cKO mESCs. Genes/TEs are ranked according to the differential expression statistic (DESeq2 Wald test; lower rank, higher expression in cKO; higher rank, lower expression in cKO). Color bar displays differential expression statistic values. (NES, normalized enrichment score). (C) Uniform manifold approximation and projection (UMAP) (integrated data) of mESCs (n = 13,349 cells). Points (cells) are colored by annotated cell state. (D) Frequency of cell states in WT and *Exosc3* cKO mESCs per biological replicate (*p < 0.05, **p < 0.005, Student’s t test; n = 3 per condition). (E) UMAP (integrated data) of mESCs. Points are colored by pseudotime (Slingshot trajectory analysis) and overlaid with RNA velocity vectors. (F) Distribution of cells along pseudotime from WT and *Exosc3* cKO mESCs, grouped by biological replicate (differences in distributions were evaluated per replicate pair by Kolmogorov-Smirnov test; representative p value indicated from replicate 1). (G) Heatmap displaying H3K27ac pileup at enhancers in mESCs. (H) TF motifs significantly enriched in the mESC-specific and exosome-sensitive enhancer clusters from (from Figure 2G). Heatmap displays the −log_10_(adjusted p value) of each target motif vs. background. (I) Aggregate signal at differential HiChIP loops (2D histogram). (J) Log_2_ enrichments (observed/expected) for the overlap of HiChIP loop anchors and enhancer clusters or 2CLC gene TSSs (***p < 0.001). (K) Correlation of differential gene and TE expression in *Exosc3* cKO and siExosc3 mESCs. (L) Schematic of experimental design for embryology experiments. (M) Bar plot displaying the number of dissected embryos containing embryonic only or embryonic and extraembryonic contribution from control siRNA and *Exosc3* KD mESCs, respectively. (N) Embryo injected with *Exosc3*-siRNAs-transfected cells showing contribution to embryonic (epiblast) and extraembryonic (VYS, visceral yolk sac) compartments. White-dotted line outlines embryonic tissues at E6.5 not covered by extraembryonic membranes. (O) Embryos injected with *Exosc3*-siRNAs-transfected cells showing contribution to embryonic (NE, neuroectoderm) and extraembryonic (VYS, visceral yolk sac) compartments. Gray-dotted line outlines the silhouette of embryonic tissues at E8.0 covered by extraembryonic membranes. Bottom panels are at higher magnifications.

Upregulation of LTR-containing TEs occurs during early embryonic development.^33^ Recently, the forced induction of MERVL expression was shown to be sufficient to revert mESCs to a more totipotent-like state,^34^ which resembles blastomeres of the 2-cell (2C) embryo.^35^ This state is conventionally referred to as 2C-like state,^35–37^ and cells acquiring this state are 2C-like cells (2CLCs). We investigated whether *Exosc3* cKO cells transcriptionally resemble 2CLCs identified and isolated by using a fluorescent reporter for MERVL and *Zscan4* expressions in mESCs.^36^ Our analysis indicates that genes specific to sorted 2CLCs are disproportionately upregulated in *Exosc3* cKO when compared with WT, as exemplified by the upregulation of MERVL and LTR-containing TEs, along with hundreds of other coding genes, ncRNAs, and pseudogenes that have been previously associated with 2CLCs (Figure 2A; Table S1). Gene set enrichment analyses (GSEAs) confirmed that a 2CLC expression signature was significantly enriched in *Exosc3* cKO compared with WT (Figure 2B). Analogous results were obtained in *Exosc3* cKO cells, compared with WT counterparts, when transitioned to a primed EpiLC state (Figures S2A and S2B).

A 2CLC state has also been observed in cells depleted for factors involved in epigenetic silencing.^38^ This supports a model in which global epigenetic repression is required to control cell fate during developmental transitions, restricting cell fate reversions such as the one from mESCs to 2CLCs. We therefore analyzed gene expression signatures of four previously published relevant datasets.^34,39–41^ Our results confirm that alteration of RNA degradation by *Exosc3* deletion is sufficient to induce a 2CLC state analogous to the one induced by the deletion of epigenetic modifiers (Figures S2C and S2D).

We further characterized the transcriptional and epigenetic features of *Exosc3* cKO using multiple omics approaches at the bulk and single-cell levels. First, we applied single-cell RNA-seq (scRNA-seq) and quantified both gene and TE expressions (see STAR Methods). As WT and *Exosc3* cKO exhibit considerable heterogeneity (Figures S2E and S2F), we conducted analyses on integrated datasets to enable equivalent cell state comparisons across genotypes. We performed graph-based clustering and supervised annotation based on marker genes to partition cells into pluripotent (ES), intermediate (INTER), and 2CLC states (Figure 2C). We found that *Exosc3* cKO led to a significant increase in the percentage of cells in the 2CLC state compared with WT (from ~3% to ~10%; Figure 2D) and a significant increase in cells in the INTER state. Pseudotime trajectory ordering augmented with RNA velocity analysis further allowed us to approximate the directionality of cell state transition (ES to 2CLC, Figure 2E). We observed a significant enrichment of *Exosc3* cKO further along the pseudotime trajectory (i.e., toward 2CLC) relative to WT (Figure 2F). This transition appears monodirectional, as we do not see evidence of cells from a 2C-like state moving to embryonic stem (ES) cell state, likely because prolonged deletion of the RNA exosome causes terminal dedifferentiation. Additionally, similar to 2C transitions in the embryo, MERVL expression and genes associated with totipotency (e.g., *Zscan4*),^42^ unlike pluripotency-associated genes (e.g., *Nanog*), increased significantly along the pseudotime progression from the ES to 2CLC state (Figures S2G and S2H). These data are consistent with a model in which suppression of RNA degradation induces an “altered” cellular state that is more amenable to transition to a 2CLC state.

The program induced during the reversion to 2CLCs is known to feature the activation of genes and regulatory elements controlled by master regulators like DUX and TP53.^43^ We thus used H3K27ac ChIP-seq data to profile enhancers, as they are key elements dictating cell state.^44,45^ We found that enhancers that are activated only in the absence of *Exosc3*, referred to as *Exosc3*-sensitive enhancers (Figure 2G), are significantly enriched for predicted binding sites for DUX, GATA, and TP53 (Figures 2H and S2I). This suggests that a 2CLC regulatory program is indeed active in *Exosc3* cKO.

To further understand the alterations in genome architecture and transcriptional regulation at the global level, we applied Hi-C with chromatin immunoprecipitation (HiChIP) to probe regions with increased or decreased spatial contacts at H3K27ac-enriched regions in WT and *Exosc3* cKO (Figures 2I, S2J, and S2K data were highly concordant with matching H3K27ac ChIP-seq; see Figure S2L). Notably, we found that in *Exosc3* cKO, the transcription start site (TSS) regions of 2CLC genes, as well as *Exosc3*-sensitive enhancers, are more associated with stronger H3K27ac loops than in WT, whereas the opposite is observed for ESC-specific enhancers (Figure 2J).

We then sought to determine whether transient loss of the RNA exosome confers an increase in cellular potency *in vivo*. To do this, we developed a system in which transient downregulation of the RNA exosome would be achieved in an embryo, as conditional deletion via tamoxifen treatment was toxic in our experimental conditions and was unlikely to work due to irreversibility of the *Exosc3* cKO-induced 2CLC state. Therefore, we first knocked down *Exosc3* with siRNA in mESCs (Figure S2M) and performed RNA-seq. Our analysis indicates that the differential expression analysis of siExosc3 vs. cKO conditions is highly concordant (Figure 2K).

To test the hypothesis that the downregulation of *Exosc3* results in the acquisition of a totipotent-like phenotype, we then performed 2C stage embryo injection of GFP-tagged mESCs that were transfected with either control scramble siRNAs or with siRNAs targeting *Exosc3*. Injected embryos were cultured *in vitro* to the blastocyst stage and then transferred into pseudo-pregnant mothers to allow for post-implantation development (Figure 2L). As expected, control mESCs contributed only to embryonic tissues but not to extraembryonic membranes (i.e., visceral yolk sac [VYS]^46,47^; Figures 2M and S2N). By contrast, cells transiently depleted of *Exosc3* were able to contribute to both embryonic and extraembryonic tissues (Figure 2M), as evident by detectable GFP signal in both embryonic epiblast and extraembryonic VYS at E6.5 (Figure 2N) and the embryonic neuroectoderm (NE) and extraembryonic VYS at E8.0 (Figure 2O).

Overall, these results show that the RNA exosome is necessary to preserve pluripotent stem cell identity and restrict a transition to a cell state associated with ERV upregulation and transcriptomic features similar to blastomeres of the 2C embryo.

### Loss of *Exosc3, Zcchc8, Rbm7*, and *Ints11* causes ESCs to acquire a 2CLC gene signature

**Figure 3.**
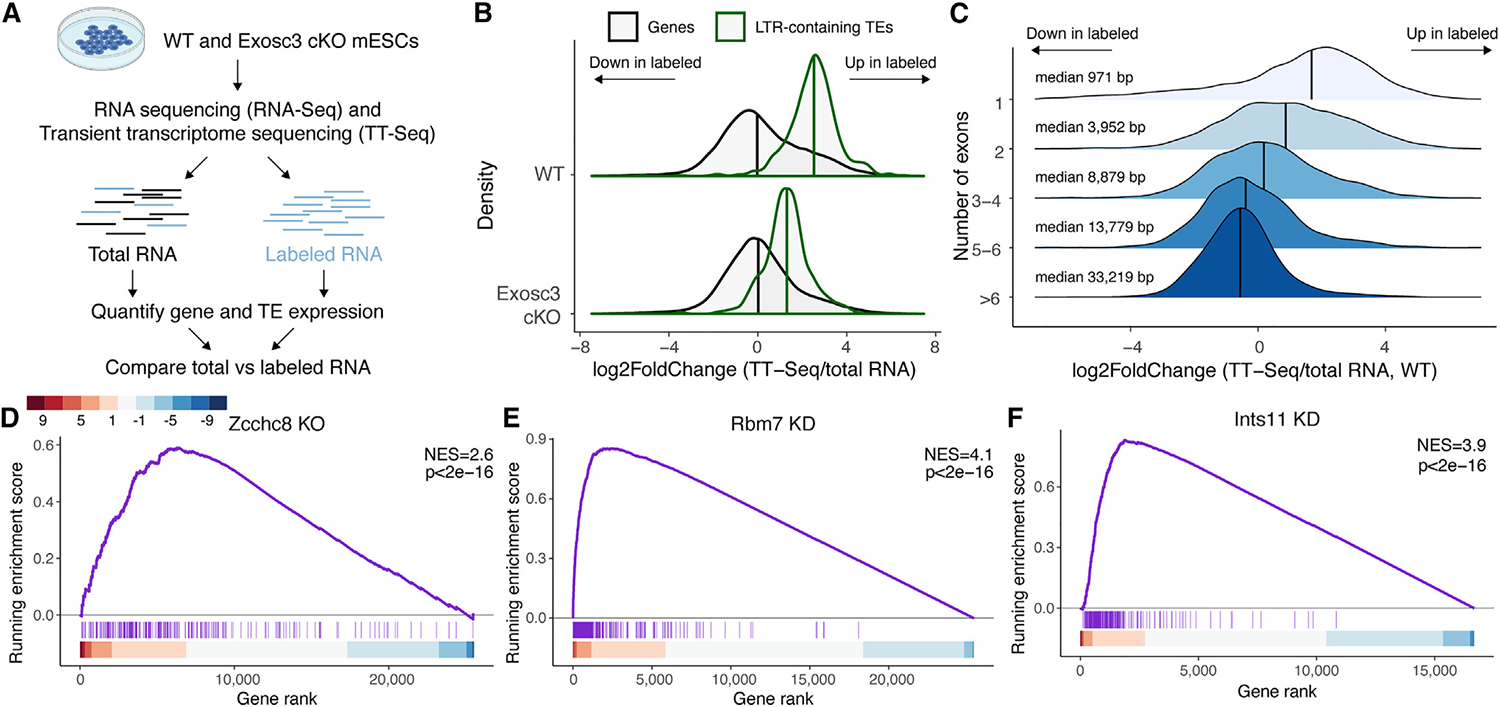
Loss of *Exosc3, Zcchc8, Rbm7*, and *Ints11* causes mESCs to acquire a 2CLC gene signature (A) Analysis workflow for the integrated analysis of bulk RNA-seq and TT-seq data. (B) Density plots displaying the distribution of log_2_-fold changes from a differential expression analysis of TT-seq labeled vs. unlabeled RNA in WT and *Exosc3* cKO mESCs. Positive-fold changes indicate higher expression in labeled RNA. (C) Density plots displaying the distribution of log_2_-fold changes of genes from a differential expression analysis of TT-seq labeled vs. unlabeled RNA in WT mESCs, grouped by the number of exons. Positive-fold changes indicate higher expression in labeled RNA. (D–F) GSEA of 2CLC genes/TEs in differential expression signatures from *Zcchc8* KO, *Rbm7* KD, and *Ints11* KD mESCs.

To decipher the mechanism by which RNA exosome-dependent RNA decay increases the likelihood of ESCs to acquire a 2CLC state, we performed a comparative analysis of the stable transcriptome and the nascent transcriptome by the metabolic labeling of RNA. To achieve this, we utilized transient transcriptome sequencing (TT-seq)^48^ in WT and *Exosc3* cKO. Labeled and total fractions were sequenced and analyzed with respect to the expressions of PCGs and ERVs (Figure 3A). As expected, labeling increased intron retention, validating the metabolic labeling procedure (Figure S3A). Our results indicate that LTR-containing TEs are enriched in nascent RNA compared with total RNA in WT (Figure 3B). In *Exosc3* cKO, the ratio of nascent RNA vs. total RNA is reduced compared with WT, indicating that more nascent LTR-containing transcripts are retained following exosome depletion. We also found differences in stability between shorter mono-exonic and longer poly-exonic genes in WT, with shorter genes displaying significantly higher enrichment in labeled RNA when compared with unlabeled RNA (Figure 3C). These results were further confirmed using an analogous metabolic labeling approach involving a short 10-min pulse with 5-ethynyluridine (EU) to minimize the 5′ bias coverage inherent to this technique^48^ (Figures S3B and S3C).

The RNA exosome is targeted to its RNA substrates by cofactors. To determine the relationship between nuclear RNA degradation and substrate specificity, we analyzed publicly available RNA-seq data from mESCs lacking components of adaptor complexes known to target the RNA exosome to nascent transcripts: the nuclear exosome targeting (NEXT) and poly(A) tail exosome targeting (PAXT) complexes. Notably, cells lacking *Rbm7* or *Zcchc8* (NEXT complex) display upregulation of MERVL and acquire a 2CLC state, whereas cells lacking *Zfc3h1* (PAXT complex) lack these phenotypes (Figures 3D, 3E, S3D, and S3E). Consistently, we find that NEXT, unlike PAXT, is downregulated in WT 2CLCs (Figure S3F). We validated these findings by performing *Rbm7* and *Zcchc8* siRNA-mediated KD in mESCs (Figures S3G and S3H). Given the known role of the NEXT complex on nascent RNA,^49–51^ these results suggest that 2CLC gene expression is regulated in a transcription-associated manner.

We then sought to determine which factors are required to coordinate RNA exosome-dependent nucleolysis. To achieve this, we performed siRNA-mediated KD of two key subunits of complexes involved in RNA cleavage and transcription termination: *Ints11* of the Integrator (INT) complex and *Cpsf2* of the CPSF complex (Figure S3I). Strikingly, 2CLC gene induction is only acquired by *Ints11* KD (Figures 3F and S3J). These data are also validated by the reanalysis of publicly available data from mESCs with reduced Ints11 achieved via siRNA or rapid degradation (Figures S3K and S3L).^52^

### Gene expression asymmetry upon loss of *Exosc3, Zcchc8, Rbm7*, and *Ints11*

**Figure 4.**
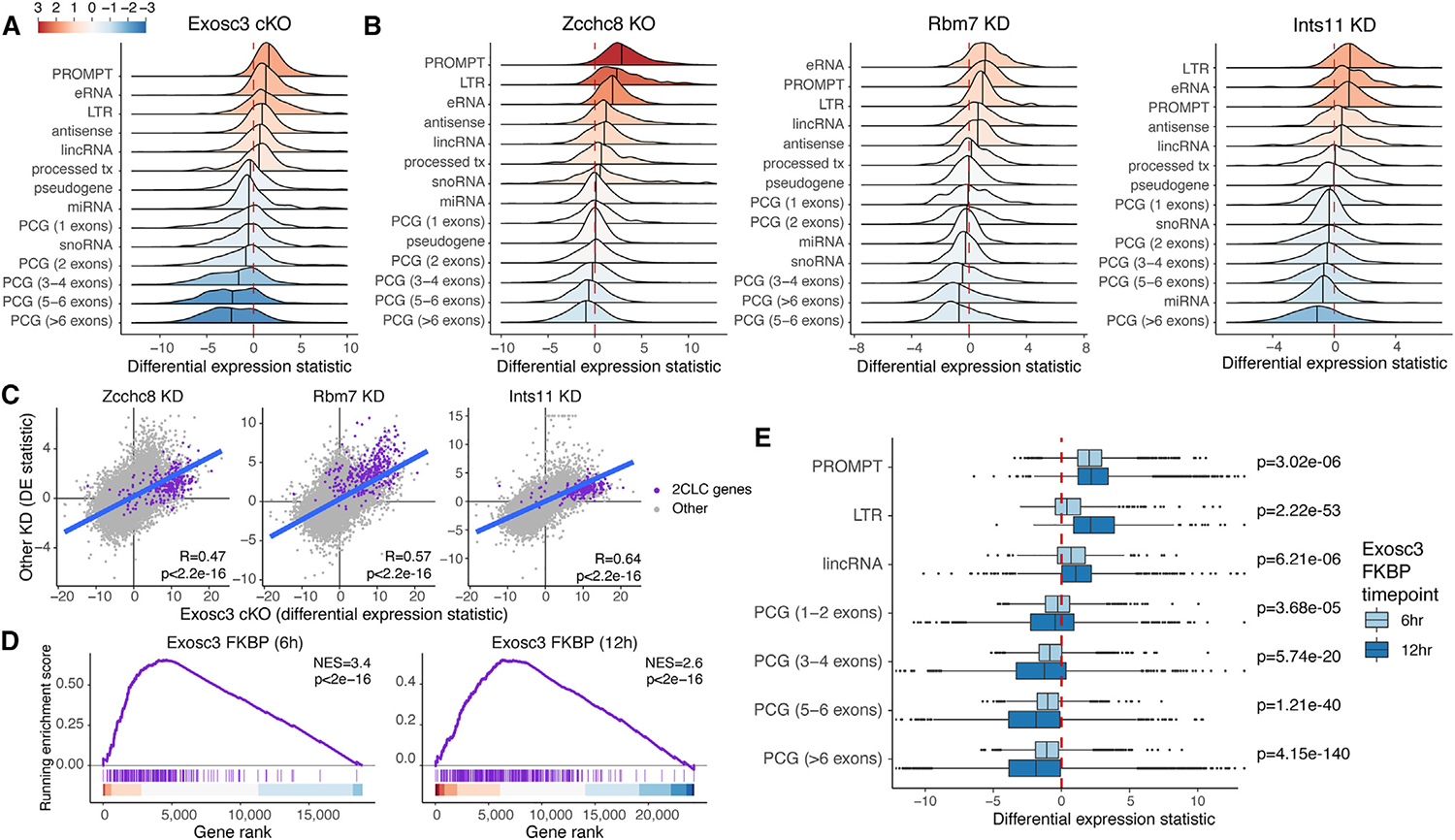
Gene expression asymmetry upon loss of *Exosc3, Zcchc8, Rbm7*, and *Ints11* (A and B) Density plots displaying the differential expression statistic (Wald test) from a differential expression analysis of *Exosc3* cKO, *Zcchc8* KO, *Rbm7* KD, and *Ints11* KD mESCs, grouped by gene biotype. Protein-coding genes are further grouped by the number of exons. Fill colors represent median differential expression statistic values per group. (C) Scatter plot displaying correlation of differential expression statistic values between WT vs. *Exosc3* cKO (x axis) and *Zcchc8, Rbm7*, and *Ints11* KD gene expression signatures (y axis) in mESCs. 2CLC genes/TEs are displayed in purple, and other genes/TEs are displayed in gray. (D) GSEA of 2CLC genes/TEs in differential expression signatures from Exosc3 rapid degradation (FKBP) in mESCs at 6 and 12 h. (E) Box plots displaying the differential expression statistic from a differential expression analysis comparing 6 and 12 h *Exosc3*-depleted mESCs for selected gene biotypes. p-values were calculated using an unpaired, two-sided Wilcoxon rank-sum test and adjusted using the Benjamini-Hochberg method (*p < 0.05, ****p < 0.0001).

In addition to the upregulation of short gene classes including LTRs and well-known targets of the RNA exosome (i.e., promoter upstream transcripts, PROMPTs; and enhancer RNAs, eRNAs, annotated *de novo* using TT-seq; see STAR Methods), we observed a strong gene-length-dependent expression signature in PCGs characterized by the downregulation of long/multi-exonic genes in *Exosc3* cKO (Figure 4A). We labeled this event as gene expression asymmetry. We confirmed that the same gene-length asymmetry is also detected upon the loss of *Zcchc8, Rbm7*, and *Ints11* (Figures 4B and S4A–S4C). Unlike long PCGs, short PCGs are largely unaffected. This is of particular interest as the INT complex has been recently suggested to have a regulatory function in premature transcription termination of many PCGs.^52^ Of note, the gene expression signatures of *Zcchc8, Rbm7*, and *Ints11* KD show a significant positive correlation with the gene signature of *Exosc3* cKO (Figure 4C), unlike those of *Zfc3h1* KO and *Cpsf2* KD (Figure S4D).

To further assess whether the observed gene-length asymmetry is a direct effect of exosome loss or rather an indirect effect due to the long-term absence of the complex, we employed a rapid degradation approach^53^ to deplete Exosc3. We genetically complemented COIN cells with FKBP-tagged *Exosc3*-expressing lentivirus. We first induced conditional ablation of endogenous *Exosc3* by tamoxifen treatment (i.e., *Exosc3* cKO), which is complemented with exogenously expressing FKBP-Exosc3, and then, we induced rapid degradation with dTAG7 treatment. Western blot revealed a near-total depletion of FKBP-Exosc3 at 12 h following depletion and partial depletion at 6 h (Figure S4E). RNA-seq of undegraded compared with Exosc3-degraded cells indicates that short-term rapid degradation of Exosc3 induces a 2CLC state (Figure 4D) and gene expression asymmetry (Figure 4E). These features start as early as 6 h post-degradation and further significantly increase between the 6 and 12 h time points.

### Premature termination defects, gene expression asymmetry, and dedifferentiation

Asymmetry in gene expression based on gene length can be due to defects in transcriptional elongation.^31,52,54^ The attenuation of long gene expression can be interpreted as transcriptional attrition due to a loss of quality control during the early phases of RNAPII transcription. A simple hypothesis that explains why gene expression asymmetry is seen upon the loss of nuclear RNA exosome (nRE) activity and by the loss of INT function is that INT and RNA exosome are epistatic, and both cleavage and degradation are steps required to prevent aberrant messenger ribonucleoproteins (mRNPs) from being licensed into productive elongation. Lack of this quality control increases the residency time on chromatin of non-productive, or prematurely terminated RNAPII, and generates transcriptional attrition.

**Figure 5.**
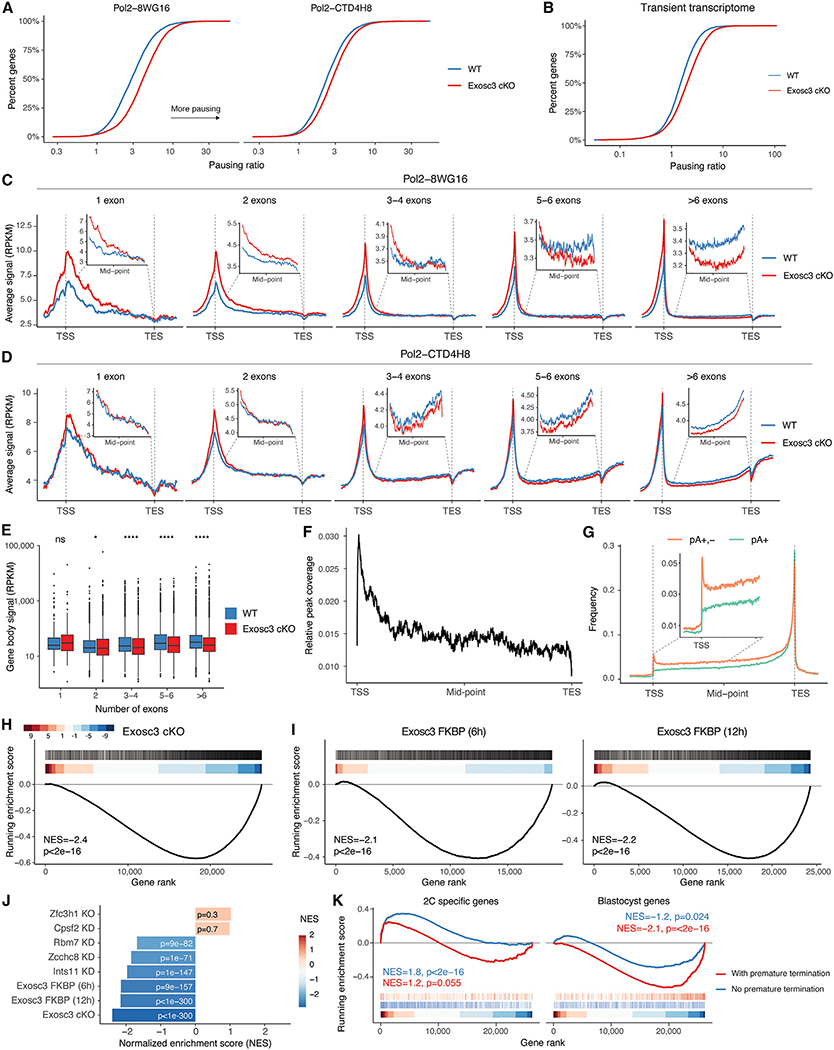
The nuclear RNA exosome controls premature termination (A) Pausing ratio of RNAPII ChIP-seq in WT and *Exosc3* cKO mESCs. (B) As above, but with metabolically labeled RNA. (C and D) Metagene plots of RNAPII ChIP-seq with 8WG16 (top) and CTD4H8 (bottom) antibodies in WT and *Exosc3* cKO mESCs, grouped by exon count. (E) Boxplots displaying normalized levels of labeled RNA in gene bodies in WT and Exosc3 cKO mESCs, grouped by exon count. p-values were calculated using an unpaired, two-sided Wilcoxon rank-sum test and adjusted using the Benjamini-Hochberg method (*p < 0.05, ****p < 0.0001). </p/>(F) Metagene plot displaying the coverage Zcchc8 eCLIP peaks relative to the transcription start site (TSS) and transcription end site (TES). (G) Metagene plot displaying the frequency of unique pA+ and pA+,− termination sites in WT mESCs relative to gene TSS and TESs. (H) GSEA of genes with premature termination events in WT mESCs, calculated in differential expression signatures derived from *Exosc3* cKO mESCs. (I) As above, but signatures derived from Exosc3-depleted mESCs by FKBP at 6 (center) and 12 h (right). (J) Bar plot displaying normalized enrichment scores (NESs) and p values (adjusted using the Benjamini-Hochberg method) from GSEA of genes with premature termination events in WT mESCs across differential expression signatures. (K) GSEA of 2C-specific genes and blastocyst genes with and without premature termination events in a differential expression signature from WT vs. *Exosc3* cKO mESCs.

To substantiate this, we profiled the pausing ratio of RNAPII by ChIP-seq, which was quantified as a ratio of promoter-proximal reads to gene body reads. Our analysis reveals that in *Exosc3* cKO, there is an increase in the accumulation of RNAPII at promoter-proximal regions, leading to a significant increase in pausing ratio, as measured with two different RNAPII antibodies (Figure 5A). These results are also substantiated by an analysis of the transient transcriptome that reveals a similar increase in promoter-proximal reads, implying a defect in productive elongation during nascent transcription (Figure 5B). Further stratification of these data by gene length reveals a gene-size-specific defect in transcriptional elongation, as significant decreases in both chromatin-associated RNAPII (Figures 5C and 5D) and nascent RNA synthesis (Figure 5E) within gene body regions are found only in genes with more than 3 exons.

The upregulation and/or resistance of expression changes in most short genes (and some long genes) upon the loss of NEXT and INT components is likely due to the following three main factors: inherently less dependence on elongation activity, facilitated re-initiation of RNAPII; and transactivation, as increased promoter strength not only results in a higher rate of gene expression but also likely renders a gene less sensitive to premature termination.

To substantiate this, we looked at an “outlier” subset of long genes that are induced in 2CLCs and which seem to elude the general rule of long genes being downregulated. Indeed, these genes are strongly induced in *Exosc3* cKO due to increased transcriptional initiation, as highlighted by the higher levels of RNAPII (Figure S5A). These include *Zscan4d*, a multi-exonic gene and known regulator of the mouse 2C state, and *P4ha2*, which features an MT2_Mm solo LTR element upstream of its TSS, consistent with previous reports that these elements play a role in regulating neighboring gene expression.^34,35,55^

To understand the relationship between nuclear RNA degradation, gene asymmetry, and a 2CLC signature, we first reanalyzed previous transcriptomic data generated in the mouse embryo,^56^ categorizing genes based on their developmental progression and by their structural features (Figure S5B). We found that 2C genes tend to be short and have lower intron count, and blastocyst genes are generally longer and multi-exonic. Of note, genes downregulated in the 2C embryo stage are the longest among the profiled categories (Figure S5C).

We then performed enhanced crosslinking and immunoprecipitation followed by sequencing (eCLIP-seq)^57^ for Zcchc8, as it was the only subunit of NEXT for which we could achieve specificity during the immunoprecipitation step (Figure S5D). Zcchc8 eCLIP enrichment is present at known targets like PROMPTs and eRNAs; notably, LTRs display a similarly high enrichment of Zcchc8 (Figure S5E). Further analysis indicated that the Zcchc8-RNA interaction tends to occur at the 5′ end of transcripts, implicating that some of the transcripts bound are nascent as previously suggested^50^ (Figure 5F). Zcchc8 eCLIP peaks are more abundant at introns, most frequently the first intron (Figure S5F). These data suggest that the newly synthesized RNA is recognized by the NEXT complex during the early phases of its transcription.

To further investigate the mechanism of action of NEXT complex, we integrated published 3P-seq data from WT and Zcchc8 KO mESCs, profiling both poly(A)+ (pA+) and poly(A)+,− (pA+,−) termination sites.^31^ In WT, we detected a peak of promoter-proximal termination detected in the pA+,−, but not in the pA+ fraction (Figure 5G). Further analysis revealed that the termination signal is directly proportional to gene length and expression level (Figures S5G and S5H). In *Zcchc8* KO mESCs, we detected an increase in the amount of pA+ termination events, especially at the 5′ end of genes, consistent with the known polyadenylation of short, pA− transcripts and handoff to PAXT in the absence of NEXT^51^ (Figure S5I). Thus, we hypothesize that long genes are more affected by premature, pA− transcriptional termination events typically cleared by NEXT-directed recognition. Reanalysis of published pA-end sequencing data in the context of exosome depletion^28,58^ further confirmed an increase of premature termination events, with this signal coming predominantly from long, highly expressed genes (Figures S5K–S5M).

Next, we investigated whether the presence of premature termination events is correlated with downregulation upon the loss of RNA degradation. GSEA revealed that genes harboring pA+,− termination sites within 500 bp of the TSS in WT are significantly negatively enriched in cells lacking Exosc3, both at 48 h *Exosc3* cKO and upon rapid degradation at 6 and 12 h (Figures 5H and 5I), suggesting that downregulation is a direct effect of RNA exosome loss rather than a secondary effect of long-term depletion. We further confirmed that genes harboring both pA+ and pA+,− premature termination events are significantly downregulated in cells depleted for *Zcchc8, Rbm7*, and *Ints11*, but not in cells lacking *Zfc3h1* or *Cpsf2*, suggesting that this effect is primarily mediated by the NEXT complex (Figure 5J). Thus, we hypothesize that RNA exosome, NEXT, and INT cooperatively function in promoter-proximal termination of RNAPII engaged with faulty mRNPs. Failure of this quality control (QC) process leads to premature termination inside gene bodies and overall manifests as elongation defects and gene downregulation, which disproportionately affect longer genes.

Finally, we sought to investigate how the lack of nuclear RNA degradation affects genes that control cell potency. As previously found, 2C-specific genes tend to be significantly shorter than late pluripotency genes (Figures S5B and S5C). Thus, we hypothesize that in cells defective of RNA surveillance, attrition and the abortive QC mechanism disproportionately affect long pluripotent genes. Notably, pluripotency genes (i.e., enriched in blastocyst) with evidence of premature termination are significantly downregulated in cells lacking *Exosc3* (Figure 5K, right) and more downregulated than those lacking such signal. On the contrary, totipotent genes (i.e., enriched in 2C) lacking premature termination signal are rapidly upregulated at early time points, whereas those with evidence of premature termination are less so (Figure 5K, left).

Taken together, this result suggests that both the downregulation of long, pluripotent genes and the upregulation of totipotent genes upon the disruption of exosome-dependent RNA decay facilitate a cellular state reversion to 2CLCs.

## DISCUSSION

In this manuscript, we investigated the relationship between RNA transcription and catabolism in early development. We discovered that RNA catabolism driven by the RNA exosome is required to elicit early quality control of RNAPII and prevent the establishment of a 2CLC gene regulatory program, characterized by gene-length asymmetry and expression of zygotic gene activation (ZGA) genes alongside specific ERVs.

We propose a model wherein transcription-associated RNA degradation is required to control gene expression in a gene size-stratified manner, establish silencing at ERV elements, and prevent embryonic cells from acquiring a transcriptional program that promotes reversion to a more primitive cellular state resembling the mouse 2C embryo.

### Transcription-coupled degradation by the RNA exosome and 2CLC

During embryonic development, ERV expression needs to be temporally and spatially regulated, as indicated by the fact that expression of different ERV classes is diagnostic of different stages of development.^59,60^ For example, MERVL expression peaks at the 2C/4C stages, whereas IAP expression is high in blastocysts and silenced in gastrulating embryos.^33^ As such, cell type-specific transcriptional programs associated with combinatorial patterns of ERV expression impose the requirement for a given silenced element (or class of elements) to become de-silenced and undergo signal-dependent activation while transitioning along the developmental axis of preimplantation development.

A unique transcriptional program is established in 2CLCs, characterized by the expression of MERVL, alongside the downregulation of pluripotent genes and upregulation of totipotent ones.^61–63^ Deletion of epigenetic modifiers controlling repressive chromatin states has been shown to result in an increase of cells with a 2CLC state, indicating that relieving silencing primes pluripotent-to-totipotent state reversion.

In this manuscript, we show that RNA exosome loss causes ES cells to transition to 2CLCs. There are many contributions to such a complex event of dedifferentiation (signaling, transcriptional effects, and gene expression effects) culminating in the establishment of a new cell identity. One main aspect is that dedifferentiation happens in cells with compromised RNA exosome activity as a result of being more receptive to 2CLC-inducing signaling. Indeed, we have shown that *Exosc3* deletion leads to a rewiring of the activity of key transcription factors that control ES reversion to 2CLC such as Dux,^41^ with Dux-dependent enhancers being activated. Although the relative contribution of all the activities generated directly and indirectly by RNA exosome deletion in the establishment of 2CLC cannot be easily parsed, performing loss-of-function experiments for factors that target the RNA exosome on chromatin simplifies our interpretations and allows us to discriminate the cellular consequences of defective RNA catabolism in ES cells (inducing a 2CLC state) from the global and gene-specific transcriptional effects.

One of the key features of the gene network established by loss of the nRE described here is the effect of RNA degradation impacting PCGs. Although the RNA exosome has been historically associated with controlling ncRNAs, some earlier evidence in *Drosophila* suggested a more pervasive role of RNA exosome on the transcription of coding genes.^64^ Our work using human cells focused on the relationship between influenza virus transcription and RNAPII activity provided further support to it.^49^ In brief, the nRE coordinates cap-snatching, the cleavage of 5′ end cellular RNA used to prime viral transcription. We showed that cap-snatching is co-transcriptional, occurs at not only non-coding but also coding cellular genes, and causes elongation defects at target genes. Based on that, we proposed that the nRE controls the transcriptional output at most genes, a conclusion that also aligns with current work performed in different cells and experimental conditions.^50,51^ In this manuscript, we show that the loss of RNA exosome at PCGs causes gene suppression in a gene size-dependent manner.

Our data imply that in the absence of RNA exosome, there is an increase in premature termination that impedes productive transcription of full-length long transcripts and manifests as a defect in elongation. Transcription is inherently an error-prone process, and there could be many initiating RNAPII engaged with aberrant mRNPs that need to be discarded. Some of these non-productive nascent transcripts are allowed to continue elongation into the downstream gene body regions upon loss of RNA exosome. However, they are more vulnerable to premature termination inside gene bodies, ultimately causing an increase in shorter unstable transcripts and a decrease in the expression of the canonical (full-length) gene. This event is bound to be dependent on gene length, as more opportunities to prematurely terminate exist in longer genes compared with shorter genes. Indeed, we detect an increase in gene downregulation as a function of gene length and a gene-size-dependent decrease in RNAPII signals over the gene body regions.

Mechanistically, the fact that gene expression asymmetry is achieved by loss of function of RNA exosome, NEXT, and INT indicates that the QC of non-productive RNAPII during early elongation is executed when the 3′ end RNAs generated by nascent RNA cleavage are degraded. Simply put, INT targets are nRE substrates. As with the RNA exosome, INT targets have historically been considered mostly ncRNAs, but recent evidence indicates a more pervasive effect of INT-dependent cleavage at most genes.^52,65,66^ Our data (1) support this latter concept, (2) reveal how degradation post-RNA cleavage of nascent transcripts is executed by the nRE, and (3) provide evidence that gene asymmetry is linked to de-differentiation. Regarding the latter point, the physiological mechanism by which cells maintain expanded potency in embryos for short temporal windows is unknown. Although inhibition of splicing has been suggested as a potential mechanism,^67^ our data imply that altering RNAPII premature termination might be another one.

*In vitro*, both mechanisms could “prime” dedifferentiation by rendering ES cells responsive to a preexistent 2C-inducing signal or by simply increasing the expression of ERVs and short genes that can pioneer cell transition to a 2CLC state while decreasing the expression of long genes (which are known to induce cell differentiation). Signaling and transactivation could potentiate ERV upregulation and the expression of long totipotent genes (that bypass suppression). In fact, among the short genes upregulated in *Exosc3, Zcchc8, Rbm7*, and *Ints11* KDs, we find potent TFs like Dux and p53 that sustain totipotency.^43^

Importantly, and in light of the many reports that delineate a clear distinction between cells resembling totipotency vs. cells that are truly totipotent,^38,68,69^ we consider the 2CLC state induced by *Exosc3* deletion as an undifferentiated terminal state, and we exclude the possibility that these cells are bona fide 2C blastomeres that can contribute to both embryonic and extraembryonic lineages.^70^ Our rationale is based on the simple genetic evidence that exosome loss of function is incompatible with life in most organisms as exemplified by the fact that conventional *Exosc3* KO mice and KO of other RNA exosome core subunits are not viable, and the *EXOSC3* gene in humans, as well as other RNA exosome core subunits genes, are loss-of-function intolerant.^7,71–73^ Unlike the loss of function of the RNA exosome that causes a terminal undifferentiated state, the transient downregulation of the RNA exosome allows cells to acquire a totipotent-like cellular identity. This is consistent with the fact that hypomorphic mutations in most exosome subunits are viable in humans. RNA exosome mutations in humans manifest primarily as neurodegenerative defects, highlighting a putative link between increased cellular potency and disease.^74^ Mutation in subunits of the INT and NEXT complex are also associated with neurodegenerative diseases.^75,76^ Notably, it has been shown that neural tissues have expression bias for longer transcripts compared with non-neural tissues.^77^ Inhibition of the resolution of torsional stress caused by transcription has been seen to affect disproportionately long genes in neurons but not in other cell types.^78,79^ This rationale might provide an insight into how defects in transcriptional regulators associated with gene length are critical particularly in neurodevelopmental and neurodegenerative disease.

### RNA-dependent regulation of TE expression

Most of our knowledge about mechanisms that silence TEs has been focused on the establishment of heterochromatic domains and/or the acquisition of repressive histone marks. Although these mechanisms likely account for most of the silencing of our endovirome in both somatic and non-somatic cells, the expansion of ERVs throughout evolution has resulted in their interspersion throughout the genome. Thus, some ERVs have been integrated in transcriptional networks controlling cell identity and differentiation. This restriction may drive the acquisition of regulatory mechanisms controlling the silencing and/or activation of some elements (or classes of elements) that are also dependent on their linear or topological genomic proximities. Critically, these structural constraints might also differ from what is conventionally referred to as heterochromatin domains.

Although heterochromatic MERVLs are associated with H3K9me2/3 silencing,^80^ our data show that a subclass of MERVL is associated with active regions of the genome in WT cells. Expression of MERVL is a key feature of the 2C state. We posit that at these elements, expression is prevented by RNA exosome-dependent degradation of spurious transcripts. When this fail-safe mechanism exerted by the RNA exosome is defective, exosome-sensitive ERVs can undergo productive transcription coupled to RNAPII elongation. ERV expression in RNA exosome KO likely depends on gene size sustained by 2C-signals and transactivation, as the latter can turn weak promoters subjected to spurious transcription into conventional promoters and sustain higher-level expression.^81^

Transcription as a prerequisite for silencing seems to be the norm rather than the exception. In fact, recent works have also established the primary role of RNA transcription in silencing of non-euchromatic ERVs, like IAP elements and LINE retrotransposons. LINE transcription is essential for both initiation and propagation of LINE repression via H3K9me3 by the HUSH complex.^82^ IAP transcription is essential for depositing H3K9me3 at IAPs via KAP1.^83^ How specificity of silencing is determined can be sequence-specific,^84^ gene structure-specific (e.g., the absence of introns as seen for LINE1),^82^ or even epigenetic modification-specific (e.g., chemical modification of transcribed RNA for IAPs).^85–87^ Future efforts to characterize ERVs as single transcriptional units will be needed to understand the complex interaction of our genome and the endovirome.

### Limitations of the study

Mapping-specific activities occurring on TEs are hampered by their repetitive nature. In this manuscript, we tried to avoid the biases introduced by using a combination of both technological and computational approaches. As unique mapping of most RNA-seq reads is impossible to achieve over long, multi-copy TEs (such as full-length MERVL, which span roughly 5 kb), we employed a computational approach that involves keeping the single best genomic alignment for each multi-mapping read, discarding all additional (i.e., secondary) genomic alignments from further analysis. We subsequently calculated the total and average signals across all genomic TE copies, thus allowing us to estimate the transcriptional and epigenetic features of these elements in cases where unique mapping was not achievable.

Our conclusions relative to the relationship between RNAPII and the nRE and INT complex are achieved using our data and the knowledge of INT and NEXT exosome as functioning in a transcription-associated manner, but we cannot exclude potential effects of RNA decay occurring differently. Although most of our experimental strategy relied on bulk analysis, better granularity could be achieved using single-cell analysis and/or *in vitro* analysis recapitulating RNAPII transcription and RNA decay interaction (chromatin template transcription).

Finally, structural variations generated by TE activity are likely a confounder in most studies that compare WT vs. mutant cells. Although we tried to bypass this problem by *de novo* genome sequencing, this strategy is far from being time- and cost-effective and cannot be performed on every cell passage and experiment.

## STAR★METHODS

### RESOURCE AVAILABILITY

#### Lead contact

Further information and requests for reagents may be directed to and will be fulfilled by lead contact Ivan Marazzi (imarazzi@uci.edu).

#### Materials availability

All unique/stable reagents generated in this study are available from the lead contact with a completed Materials Transfer Agreement.

#### Data and code availability

- Raw and processed sequencing data have been deposited at GEO under accession GEO:GSE205211. All data are publicly available as of the date of publication.
- This paper does not report any original code.
- Any additional information required to reanalyze the data reported in this paper is available from the lead contact upon request.

### EXPERIMENTAL MODEL AND STUDY PARTICIPANT DETAILS

#### Cell culture

*Exosc3* Cre/lox conditional inversion (COIN) mouse pluripotent stem cells were gifted from the Uttiya Basu lab. mESCs were cultured in N2B27 media consisting of a 1:1 mixture of DMEM/F12 with Hepes (Gibco, 11330032) and Neurobasal (Gibco, 21103049) supplemented with 1X N2 (Gibco, 17502058), 0.5X Serum-free B27 (Gibco, 17504044), 1X L-Glutamine (Gibco, 25030081), 1X Antibiotic-antimycotic (Gibco, 15240062), 0.05% Bovine Albumin Fraction V (Gibco, 15260037) and 1X 2-Mercaptoethanol (Gibco, 21985023). For naïve mESC, 3 μM CHIR99021(Reprocell, 04–0004), 1 μM PD03255901 (Reprocell, 04–0006), and 20 ng/mL mouse recombinant leukemia inhibitory factor (LIF) (R&D systems, 8878-LF-500/CF) were added to the media to sustain stemness. For EpiLC differentiation, naïve mESC were treat with Accutase (Corning, 25–058-CI) for 2–5 minutes in a 37°C incubator to obtain a single cell suspension. Approximately 100,000 cells per well were plated in 12-well plate coated with 10 μg/mL Human plasma fibronectin (EMD Millipore, FC-010–5mg) in N2B27 media supplemented with 1% KnockOut serum replacement (Gibco, 10828028), 20ng/mL Activin A (R&D systems, 338-AC-050/CF), and 12ng/mL Fgf2 (R&D systems, 233-FB-025). To induce *Exosc3* conditional inversion, 100nM 4-Hydroxytamoxifen (EMD Millipore, 508225) was added the day after mESC were passaged or the day of EpiLC differentiation. Media was washed out the next day and changed daily. Cultures were discarded after 20 passages. Cells were collected for experiments after 48 hours. For differentiation protocols requiring mESC in serum LIF conditions, cells were grown in KnockOut DMEM (Gibco, 10829018) supplemented with 20% KnockOut serum replacement (Gibco, 10828028), 1X L-Glutamine (Gibco, 25030081), 1X Antibiotic-antimycotic (Gibco, 15240062), 1X Sodium pyruvate (Gibco, 11360070), 1X MEM Non-Essential Amino Acids Solution (Gibco, 11140050), 1X 2-Mercaptoethanol (Gibco, 21985023), and 20 ng/mL mouse recombinant leukemia inhibitory factor (R&D systems, 8878-LF-500/CF).

### METHOD DETAILS

#### Bulk RNA extraction and cDNA synthesis

For RNA extraction, cells were washed 3x with DPBS and lysed directly on the plate with 1mL of TRIzol reagent (Invitrogen, 15596026). Subsequently, RNA was purified using Direct-zol RNA Miniprep Plus Kits (Zymo, R2072) following manufacturer instructions. In brief, lysed cells in TRIzol were mixed 1:1 with 100% ethanol. Mixed lysates were added to the Zymo-spin columns, treated with DNase I, and washed with provided buffers. RNA was eluted with UltraPure DNase/RNase-Free Distilled Water (Invitrogen, 10977015). For complementary DNA synthesis, 100–200 μg of DNase I treated RNA was reverse transcribed using High-Capacity cDNA Reverse Transcription Kit (Applied Biosystems, 4368814). Random hexamers were used to capture total RNA. For metabolic RNA labeling, cDNA synthesis was carried out while RNA was bound to beads using SuperScript VILO cDNA synthesis kit (Invitrogen, 11754–050).

#### Illumina RNA library preparation and sequencing

Purified RNA was submitted to the Icahn School of Medicine Genomic Core facility for sequencing. Paired-end libraries were prepared with Illumina Stranded Total RNA Prep with Ribo-Zero Plus kits (Illumina, 20020596). Libraries were sequenced on an Illumina NovaSeq 600 S2 instrument with 50 million 2×125bp reads per sample.

#### Chromatin immunoprecipitation

##### H3K9me3

Approximately 1×10^7^ control and *Exosc3* cKO pluripotent stem cells were dissociated from culture and fixed in 50 mL of N2B27 media with 1% methanol free formaldehyde for 10 minutes at room temperature. Fixation was quenched using .125 M glycine for 5 mins at room temperature. Pellets were then washed 3x with ice-cold DPBS supplemented with Halt protease and phosphatase inhibitors (Thermo Scientific, 78446). Pellets were frozen in −80°C overnight. Frozen pellets were then thawed on ice and 10×10^6^ cells were lysed with 1 mL of LB1 (50 mM Hepes-KOH pH 7.5, 140 mM NaCl, 1mM EDTA, 10% glycerol, 0.5% NP-40, 0.25% Triton x-100) for 20 minutes rotating in 4°C. Chromatin was pelleted and supernatant was then discarded. Nuclear envelope of the chromatin fraction was lysed with 1 mL LB2 (Tris-HCl pH 8, 200 mM NaCl, 1 mM EDTA, 0.5 M EGTA), rotating for 10 minutes at room temperature. Pellets after LB2 lysis were resuspended in 300 μL of LB3 (Tris-HCl pH 8, 100 mM NaCl, 1 mM EDTA, 0.5 M EGTA, 0.1% Na-Deoxycholate, 0.5% N-lauroylsarcosine). Chromatin was sonicated in LB3 was aliquoted in 100 μL increments in micro AFA Fiber Crimp-Cap 6×16mm tubes (Covaris, 520091). Chromatin was sheared on a Covaris instrument with peak power set to 450 for 700 seconds. Aliquots of sheared chromatin were pooled for immunoprecipitation. Antibody was coupled to Protein A Dynabeads (Invitrogen, 10001D) for 4 hours rotating in 4°C. Coupled beads were washed 3x with 0.05% BSA in DPBS. Antibody bound beads were then added to chromatin in LB3 overnight rotating in 4°C. Beads were washed 8 times with RIPA buffer (500 mM Hepes-KOH pH 7.6, 100 mM LiCl, 0.5 M EDTA, 1% NP-40, 0.7% Na- Deoxycholate). An additional wash with TE buffer (10 mM Tris-HCl pH 8, 1 mM EDTA, 50 mM NaCl) prior to elution. Bound chromatin was eluted in elution buffer (50 mM Tris-HCl pH 8, 10 mM EDTA, 1% SDS) shaking for 30 minutes at 65°C. Decrosslinking was achieved by the addition of 8 μL 5 M NaCl to 210 μL of eluted chromatin. RNA and protein were digested by using 500 μg/mL RNase (Invitrogen, AM2271) and 22 mg/mL proteinase K (Invitrogen, AM2546) for 2 hours at 55°C. DNA for library preparation and sequencing was purified using MinElute PCR Purification Kit (Qiagen, 28004).

##### H3K27ac and Rbp1

Cell fixation and quenching were done exactly as described above. Approximately 10×10^6^ cells were lysed for 15 minutes on ice in 500 μL of cell lysis buffer 1 (10 mM Tris-HCl pH 8, 10 mM NaCl, 0.2% NP-40). Nuclei were pelleted and subsequently lysed in 500 μL of nuclear lysis buffer (50 mM Tris-HCl pH 8, 10 mM EDTA, 1% SDS) for 10 minutes on ice. Chromatin was then sheared using the Diagnode Bioruptor Plus for 30 cycles set to 30 seconds on and 30 seconds off. Sheared chromatin was then precleared using species specific IgG for 2 hours at 4°C. Antibodies for protein of interests bound to chromatin were couple to Protein A Dynabeads (Invitrogen, 10001D). Coupled beads were added to precleared chromatin and rotated overnight at 4°C. Beads bound to chromatin were then resuspended with 1 mL of IP wash I buffer (20 mM Tris-HCl pH 8, 2 mM EDTA, 50 mM NaCl, 1% Triton x-100, 0.01%SDS). Beads were bound to a magnetic stand and washed twice with a high salt buffer (20 mM Tris-pH 8, 2 mM EDTA, 500 mM NaCL, 1% Triton x-100, 0.01% SDS0 and once with IP wash II buffer (10 mM Tris ph8, 1 mM EDTA, 0.25 M LiCl, 1% NP-40, 1 % Na- Deoxycholate). DNA was eluted twice by applying 100 μL of elution buffer (1.25% SDS, 100 mM NaHCO_3_) to each sample and incubating at 65°C shaking at 800 rpm. Decrosslinking and DNA purification were identical to the steps described above. Paired-end libraries from purified DNA were prepared using NEBNext^®^ Ultra II DNA Library Prep Kit for Illumina (NEB, E7645S) per manufacturer recommendations. Size selection of DNA prior to library was not performed. Libraries were sequenced on a NextSeq 550 instrument with a goal of 2×75bp 30 million reads for each sample.

##### HiChIP

HiChIP^106,107^ data was generated by Arima Genomics using the ArimaHiC+ kit (A101020), according to the manufacturer’s protocols.

#### ATAC-Seq

For ATAC-Seq, a modified version of the Omni-ATAC^108^ was performed as previously described.^109^ Approximately 50,000 mESCs were used for each reaction. ATAC-Seq library preparation was performed as previously described.^110^ Multiplexed libraries were sequenced on a NovaSeq 6000 instrument with a 75bp paired-end reads and a goal of 50M reads per sample.

#### Genomic DNA extraction

##### PacBio genome sequencing

For DNA extraction, PureLink^™^ Genomic DNA Mini Kit (Invitrogen, K1820–00) was used following manufacturer instructions. In brief, cells were washed 3x with DPBS and lysed directly on the plates using supplied Lysis/Binding buffer. Lysates were subsequently applied to the column and washed with buffers provided. DNA was eluted in PureLink Genomic Elution Buffer provided in the kit. DNA was sequenced using Pacific Biosciences (PacBio) CLR SMRT sequencing.

##### WGBS

Genomic DNA was isolated from two WT mESCs clones and two cKO clones using a Quick DNA kit (Zymo Research). Bisulfite conversion and generation of sequencing libraries were performed by GENEWIZ (Azenta Life Sciences). The libraries were sequenced on an Illumina HiSeq 2500 sequencer as 150-bp paired-end reads.

#### 10X single-cell RNA sequencing

Triplicate wildtype or Exosc3-KO samples in single-cell suspension were each independently labeled with MULTI-seq sample barcode lipid-modified oligonucleotides (kindly provided by Zev J Gartner, University of California, San Francisco), as previously described.^111^ After labeling, cells from each of the six total samples were pooled at equal concentrations, and processed for single-cell RNA sequencing in accordance with the Chromium Next GEM 3′ v3.1 protocol (10x Genomics) for a targeted cell recovery of 20,000 total cells. MULTI-seq libraries were prepared as previously described.^111^ Both gene expression and MULTI-seq libraries were sequenced on an Illumina NextSeq 500 instrument to an average of 41,304 reads per cell for gene expression libraries and average of 8,770 reads per cell for MULTI-seq libraries.

#### Chimeric contribution

Karyotypically normal mouse ES cells were routinely cultured in 2i-LIF and passaged at a 1:6–1:8 ratios. ES were at passage 14–16 were infected with a lentivirus expressing GFP and PURO ubiquitously. ES cells were treated with Puromycin for two weeks (2ug/mL) to obtain a population expressing homogenously high levels of GFP. GFP-ES cells were electroporated with siRNA targeting Exosc3 or control Scramble siRNAs and replated for 24 hrs. After 24 hrs ES cells were microinjected (15–20 cells per embryo) into 2C mouse embryos (CD1). Injected embryos were then cultured to blastocyst stage in vitro and transferred to pseudopregnant females making sure to transfer between 8–10 embryos per uterine horn. Pseudopregnant females were then euthanized between E6.5 and E8.0 and embryos analyzed for chimeric contribution.

#### Nascent RNA capture

Metabolic labeling for nascent RNA capture was carried out using Click-iT Nascent RNA Capture Kit (Life Technologies, C10365) following manufacturer instructions. Briefly, control and Exosc3 cKO mESC were cultured in 1 mL of 2i/LIF media for 10 minutes supplemented with 0.5mM 5-ethynyl Uridine (EU). Media was subsequently washed out 3x with DPBS prior to lysis with TRIzol. Approximately 500 ng of extracted RNA was biotinylated with 0.25 mM biotin azide. EU labeled RNA were bound to Dynabeads MyOne Streptavidin T1 (Invitrogen, 65601). Bound RNA was washed on a magnetic stand to purify only EU incorporated RNA using provided wash buffers. Bound RNA was then reverse transcribed as described above. TT-Seq library construction was performed as previously described^48^ in the Nascent Transcriptomics Core at Harvard Medical School, Boston, MA.

#### FKBP-Exosc3 degron experiments

The coding sequence for full length mouse *Exosc3* was N-terminally tagged with 3xFlag-FKBP12^F36V^ and inserted into a modified pLVX-puro lentiviral vector to generate pLVX-Puro_mmExosc3-FKBP. For lentivirus generation, HEK293T cells were seeded to 50% confluency one day prior to transfection with pLVX-puro_mmExosc3-FKBP, packaging vector psPAX2 and envelope plasmid pMD2.g (gift from Didier Trono lab) in the ratio of 5:5:1. Lipofectamine 2000 reagent was used for transfection following the manufacturer’s instructions. 18h post transfection, the media was changed, and fresh media added to transfected cells. Subsequently, cell culture supernatant was collected at both 24h and 48h post infection. Supernatant were clarified by centrifugation for 1500rpm, 5min at 4°C and filtered through a 0.45μm filter. Lentiviral particles were then concentrated 100X using the Lenti-X concentrator reagent (Takara) following the manufacturer’s protocol.

To generate the FKBP-Exosc3 COIN cell line, COIN WT-mESCs were infected with concentrated lentiviral particles described above at low multiplicity of infection such that ~10% cells are infected. 48h post infections, cells were dissociated using accutase, and replated into N2B27 + 2i/Lif media containing 0.5μg/mL puromycin. A control well containing uninfected cells in puromycin containing media. Selection was considered completed when cells in the control well were all dead. FKBP-Exosc3 COIN cells were then expanded for further experiments.

For degron experiments, 5e4/well FKBP-Exosc3 COIN cells were plated onto gelatin coated 48 well plates, and grown in N2B27 + 2i/Lif in the presence of 100nM Tamoxifen for 48h. Ethanol treated control wells were also included. 48 hours post tamoxifen or ethanol treatment, media was aspirated and replaced by fresh N2B27 + 2i/Lif media without tamoxifen or ethanol. dTAG-7 (Sigma) reagent or DMSO control (0h) was then added to a final concentration of 500nM at 6h or 12h prior to sample collection. Final collection of all samples was done at 72h from the first addition of Tamoxifen/Ethanol. All conditions were done in triplicate.

For RNA-seq library generation, cells were collected in 250μL Trizol reagent, and RNA was isolated according to the manufacturer’s instructions. DNA was removed from RNA samples using the Turbo DNA-free kit. RNA-sequencing libraries were generated using NEBNext^®^ Ultra^™^ II Directional RNA Library Prep Kit for Illumina^®^ and NEBNext^®^ rRNA Depletion Kit (Human/Mouse/Rat) following manufacturer’s protocol. Final pooled libraries were sequenced paired-end (2×75bp) on the NextSeq500 (Illumina).

To verify protein KD in the same samples used for RNA-Seq, protein was isolated from the trizol organic phases of samples post-choloroform extraction following the manufacturer’s protocol. Protein pellets were resuspended in 1X LDS buffer containing reducing agent and boiled for 15 min at 70C. Protein extracts were then ran on a 1.5mm 4–12% NuPAGE Bis-Tris gel at 150V for 75min. Proteins were then transferred onto a 0.22μm nitrocellulose membrane (Amersham) (30mA, 2hours). After transfer, membranes were washed 1x in PBS, blocked in 3% milk in TBST (TBS + 0.1% Tween) for 30min at RTP. Membranes were then incubated at 4°C overnight in with 1:1000 diluted anti-Exosc3 antibodies (Abcam; ab156683) in 3% milk in TBST. Membranes were then washed three times (5min, RTP, rotating) in TBST, before stained with secondary antibody (anti-Rabbit-HRP, CST, Cat#: 7074S, 1:5000) in 3% milk/TBST. Membranes were then washed a further three times in TBST (5min, RTP, rotating) before being probed with ECL (Clarity ECL Western Substrate, Biorad) and imaged using film. To visualize Flag-tagged FKBP-Exosc3 or β-Actin loading controls, membrane were incubated 2h at RTP in 3% milk/TBST containing either 1:2500 diluted anti-Flag-M2-HRP (Sigma, A8592-.2MG) or 1:2500 diluted anti-B-Actin(8H10D10)-HRP (CST; 12262S).

#### Zcchc8 eCLIP

eCLIP experiments were performed as previously described in Van Nostrand et al.,^57^ but with the following modifications: ~3.8E7 COIN WT-mESC cells grown in 6 well dishes coated with gelatin. At time of collection, cells were dissociated into a single cell suspension with Accutase at 37°C for 5min and transferred into a 50mL falcon tube. Accutase was then diluted out with 10X volume N2B27 media and cells were spun down at 300g for 3min at 4°C. Cells were then resuspended in 15mL ice cold PBS and transferred to a 15cm dish. Cells were then UV-crosslinked (400mJ). Cross-linked cells were then collected, and spun down at 300g for 3min at 4°C. Supernatant was removed, and the cell pellets were flash frozen in liquid nitrogen and stored at −80°C.

On day of lysis, frozen cell pellets were resuspended in ice cold iCLIP lysis buffer (50mM Tris-HCL pH7.5, 100mM NaCl, 1% Igepal CA630, 0.1% SDS, 0.5% Sodium deoxycholate and 1.1% Murine Rnase Inhibitor (M0214L, NEB)) at a density of 2E7 cells/mL. Cells were lysed for 15min on ice, before a short sonication (3min, 30 sec on/ 30 sec off; low setting) in the Diagenode Bioruptor Plus. Sonicated cell lysates were then treated with 40U of RnaseI (Ambion) and 4U of Turbo Dnase (Ambion) for exactly 5min at 37°C. Nuclease activity was then stopped with the addition of 1% SuperaseIn reagent. Cell lysates were then clarified by centrifugation (15000g, 15min, 4°C). 800μL of lysate was used for each IP reaction. Two IPs were performed per condition, using either 10μg anti-Zcchc8 antibodies (Proteintech, Cat #: 23374–1-AP, Lot: 00076174), or 10μg control Rabbit IgG (polyclonal) (Abcam, Cat #: ab37415, Lot: GR3327091–1). Antibodies were pre-bound to Protein A Dynabeads (Thermo fisher scientific, Cat: 10001D) prior to IP. 16μl of lysate was saved for 2% input before addition of antibody-bead complexes. Immunoprecipitation was performed with rotation, at 4°C overnight.

The next day, immunoprecipitated Protein-RNA complexes were ligated to 3’RNA linkers on-bead, following wash steps and a dephosphorylation/phosphorylation step using FastAP and PNK in the presence of SuperaseIn Rnase inhibitors. Protein-RNA complexes were then eluted from beads in 1X NuPAGE^™^ LDS sample Buffer (Thermo fisher scientific), containing Reducing agent (Thermo fisher scientific), at 70°C for 10 min. An aliquot of this material was also used in a separate western blot to confirm Zcchc8 migration position as well as specificity of the IP. Protein-RNA complexes were then ran on a 1.5mm 4–12% NuPAGE Bis-Tris gel (Thermo fisher scientific) at 150V for 75min. Protein-RNA complexes were then transferred onto a 0.22μm nitrocellulose membrane (Amersham) (30mA, 2hours). After transfer, membranes were washed once in ice cold PBS. Regions corresponding to approximately 10KDa below and 75KDa above Zcchc8 bound complexes were then excised using a clean scalpel. Excised membrane pieces were sliced into 1mm strips, and subject to proteinase K digestion. RNA was isolated using Acid Phenol/Chloroform (pH 4.5) (Ambion), and further cleaned up using the Zymo RNA Clean and Concentrator kit (Zymo).

Extracted RNA was then reverse transcribed. Following 5′ linker ligation, linker ligated cDNA was cleaned up and amplified using 14 cycles PCR. The final library was purified using 1.8X Ampure XP (A63881, Beckman Coulter) beads according to manufacturer’s recommendations. Adapter dimers were removed with a second bead selection step using 1.4X bead to eluate ratio. Library concentration was checked with the Qubit, and sizing (150–300bp) checked with the DNA high sensitivity Bioanalyzer chip. Libraries were pooled and sequenced paired-end (2 × 75bp) using the Illumina NextSeq500.

### QUANTIFICATION AND STATISTICAL ANALYSIS

#### Bulk Illumina RNA-seq analysis

Illumina adapters were trimmed from reads using Trim Galore (v0.5.0).^112^ Reads were aligned to the GRCm38/mm10 reference genome using STAR (v2.7.5b)^88^ with the following custom parameters: –outFilterMultimapNmax 100 –winAnchorMultimapNmax 100. Gene and TE-level counts were calculated using TEcount (v2.2.1).^89^ Gene expression quantification and subsequent downstream analyses were performed using a custom reference transcriptome based on the GRCm38 v102 Ensembl annotation, publicly available at GEO:GSE205175 (results were consistent with quantifications using Ensembl alone). For TEs, the GRCm38 RepeatMasker annotation from UCSC table browser was used. Publicly available data from the following GEO series was integrated and processed as described above: GEO:GSE75751,^36^ GEO:GSE119819,^34^ GEO:GSE85627,^41^ GEO:GSE94556,^40^ GEO:GSE69484,^39^ GEO:GSE115727,^32^ GEO:GSE178550,^31^ GEO:GSE200702.^52^ Differentially expressed genes and TEs were calculated using DESeq2 (v.1.30.0)^90^ in and R 4.0.3. environment. P-values were adjusted using the Benjamini-Hochberg method. Gene set enrichment analysis was performed using fgsea (v1.16.0)^91^ ranking genes according to the differential expression statistic output by DESeq2. The 2CLC gene set was determined from GSE75751,^36^ using genes and TEs that are upregulated in MuERVL+/Zscan4+ double positive samples when compared to untransfected negative control samples (FDR < 0.05 and log2FoldChange > 5). Gene length stratification was performed by selecting the major isoform for each gene using StringTie (v2.2.0).^94^ Gene clusters from mouse preimplantation embryo RNA-Seq (GEO:GSE138760, Figures S5B and S5C) were identified using Mfuzz (v2.50.0).^95^

#### PacBio genome sequencing analysis

Structural variations in the *de novo* sequenced ES cell genome compared to mm10 were identified using PBSV. Repetitive element content of the newly identified structural variations was determined by running RepeatMasker (v4.1.1) on the nucleotide sequence of insertions and deletions using default parameters. Dot plots depicting MERVL insertions and deletions were generated using MacVector (v18.1).

#### WGBS analysis

Quality- and adapter-trimmed raw sequences were aligned to the GRCm38 primary genome and deduplicated using bismark.^113^ CpG methylation data extracted with the bismark_methylation_extractor function were modified to construct a bsseq object.^114^ Only CpG sites covered at least 6 times in all four samples (two clones per genotype) (n= 19,027,274) were considered for the downstream analyses. There were 10.7×10^6^ CpG sites in gene regions and 6.5×10^6^ sites in the TE regions meeting this condition. Genic and TE regions were extracted using the UCSC table browser (GENCODE VM23 knownGene and rmsk, respectively).

#### ChIP-seq and ATAC-Seq analysis

Adapters were trimmed from reads using Trim Galore.^112^ Reads were mapped to mm10 using bowtie2 (v2.4.1)^96^ with default parameters. BAM files were filtered using sambamba (v0.5.6),^97^ removing reads that are duplicated, unmapped, and removing all secondary alignments (i.e. keeping the best genomic alignment location for multimapping reads). BigWigs were generated on the filtered BAM files using deepTools (v3.5.0)^115^ with RPKM normalization. To calculate the enrichment of ChIP-Seq data across repetitive elements, RepeatMasker annotations for mm10 were first downloaded from the UCSC Genome Browser and filtered by requiring at least 50 genomic copies of at least 50bp in length. Next, the average coverage of each ChIP-Seq dataset across each TE copy in the genome was calculated from the BigWig files using the multiBigWigSummary function from deepTools. A random background signal was then calculated by using the same approach on randomly shuffled TE sequences using bedtools. Enrichment of TEs was then computed for every ChIP-Seq dataset by calculating the ratio between the average values of observed and background signal across all genomic copies of each element. Stratification of TEs in Figure 1F was performed using a cutoff of log2FoldChange>0.5 and adjusted p-value<0.05 when comparing TE ChIP-Seq signal versus background. Heatmaps were generated using deepTools. ChIP-Seq data for MPP8 was downloaded from GEO:GSE178550^31^ and processed as described above. Enhancers were identified from H3K27ac data using ROSE.^116,117^ Motif analysis on enhancer regions was performed using HOMER.^104^

#### PROseq and CAGE analysis

PROseq and CAGE data was downloaded from GEO:GSE115727 and processed as previously described.^32^ PROseq adapter sequences were removed using Trim Galore, filtering for a minimum 15bp read length, and reverse complemented using seqkit (v0.10.1)^99^) Reads were first mapped to a copy of the mouse rDNA repeat (GenBank: BK000964.1) using bowtie2, and unmapped reads were aligned to the mouse mm10 genome. CAGE reads were trimmed by 11bp from the 5’ end to remove linker sequences and by 11bp from the 3’ end, and subsequently aligned to the mouse mm10 genome using bowtie2. Prior to visualization, PROseq and CAGE BAM files were filtered by removing unmapped reads and secondary alignments. Profile plots were generated using deepTools.

#### scRNA-seq data processing

Sequencing data were processed with CellRanger (v5.0.1) (10X Genomics, Inc). Cellranger mkfastq was used to convert bcl image sequence files into fastq files for all gene expression and MULTI-seq libraries. Reads (gene expression libraries) were aligned and quantified to the mouse reference genome (mm10; Ensembl v98) with STARsolo v2.7.5b.^118^ Output BAM files were processed with the scTE package (v1.0.0)^92^ to generate gene-cell matrices containing both annotated genes and transposable elements/endogenous retroviral viruses (TE/ERV) were quantified. A cell barcode whitelist derived from the CellRanger count-generated filtered gene-cell count matrices was used to filter the scTE-processed gene-cell count matrices. Reads from MULTI-seq libraries were quantified against a sample barcode reference with CellRanger count using the “Antibody Capture” library setting. Filtered gene-cell count matrix (annotated gene expression/TE/ERV) and sample barcode-cell matrix (MULTI-seq) were then analyzed with Seurat (v4.0.1).^93^ Sample demultiplexing was performed using Seurat’s HTODemux function. After an initial filter to remove cells with fewer than 8,000 unique molecular identifiers (UMIs), more than 40,000 UMIs, or greater than 10 percent mitochondrial gene expression; cells classified as ‘singlets’ by HTODemux were maintained for downstream analyses (13,349 cells total post-filter).

#### scRNA-seq data analysis

Data were first normalized using Seurat’s NormalizeData function (default settings), and cell cycle scoring (S- and G2M- phase scores) was performed using Seurat’s CellCycleScoring function. Each dataset was then independently normalized with SCTransform (variables.to.regress set to cell cycle scores (S and G2M) and percent mitochondrial gene expression); highly variable gene selection was restricted to annotated genes (i.e. excluding TE/ERV). Datasets were then integrated with Seurat’s PrepSCTIntegration, FindIntegrationAnchor and IntegrateData functions (default settings). Principal component analysis was conducted on the integrated data object and the first 30 principal components were selected for unsupervised graph-based clustering (resolution: 0.4) and uniform manifold approximation projection visualization. Clusters were annotated and grouped into 3 major cell states in a supervised manner using canonical embryonic stem cell and ‘2c-like’ stem cell markers. “Pluripotency” and “totipotent/2c-like cells” gene lists were curated from the literature^67^ and were used as input for Seurat’s AddModuleScore function to calculate gene set scores. Trajectory and lineage reconstruction analysis of the integrated data was performed with the slingshot package v1.8.0.^119^ To test for differences in cellular progression/differentiation along the identified trajectory between the Exosc3−/− and Exosc3WT genotypes, we estimated cell densities for each genotype across pseudotime and performed the Kolmorogov-Smirnov test to ascertain if the genotype-sample densities were derived from the same distribution. RNA velocity analysis was performed using the velociraptor (v1.0.0) package (https://github.com/kevinrue/velociraptor). RNA velocity vectors were calculated using velociraptor’s scvelo function with the inference mode set to ‘dynamical’. Calculated velocity vectors were embedded and visualized by UMAP.

#### HiChIP data processing

HiChIP reads were aligned and processed using HiC-Pro.^103^ FitHiChIP was then used to identify chromatin loops separately from WT and cKO samples, using loop calling parameters for peak-to-all and FitHiChIP(L) background modeling.^105^ Merging redundant loops across experiments yielded a total of 123,354 loops. To identify loops with increased strength in WT or cKO, raw interacting read counts from each replicate experiment and then normalized and variance stabilized using DESeq2’s VST function. Loops with an average difference greater or less than 0.15 were used to define the most strongly induced (n=2292) or weakened (n=3589) in cKO relative to WT. Loop anchors, defined at a resolution of 5 kb, were assessed for their genomic overlap with various featues (2C gene TSS, DUX peaks, etc.) using HOMER’s mergePeaks command. APA plots were created by first converting valid pairs from HiC-Pro to cooler format and then using hicExplorer’s hicAggregateContacts tool to generate the APA plots.^120^

#### Metabolically labeled RNA-seq analysis

TT-Seq and EU-Seq reads were trimmed, aligned, and filtered as described in the bulk Illumina RNA-Seq analysis section. Splicing efficiency was determined using SPLICE-q.^121^ Strand-specific BigWig files were generated using deepTools with RPKM normalization. Transcriptional Units (TUs) corresponding to regions of continuous transcription were defined from the normalized TT-Seq files using GenoSTAN (v2.18.0)^100,122^ In order to identify TUs corresponding to PROMPTs and eRNAs, several filters were applied. First, regions deriving from transcription of annotated genes were filtered by removing TUs fully containing at least one known transcript on the same strand, as well as TUs whose sequence substantially overlaps with that of known transcripts on the same strand (at least 3 Kb, or at least 25% of the total TU length). To identify eRNAs, filtered TUs were further refined by requiring a proximity within 500bp of enhancers (de-novo annotated using ROSE, see STAR Methods above). To identify PROMPTs, filtered TUs were further refined by requiring proximity within 1 Kb of an annotated TSS in the antisense strand.

#### Pausing ratios

Pausing ratios were defined by first calculating the normalized signal in promoter-proximal regions (−30 bp TSS + 300bp) and gene body (TSS+300bp to TES). Normalized signal was calculated using the multiBigwigSummary function from deepTools. Genes were filtered by requiring non-zero signal in both regions. For ChIP-Seq data, genes were further filtered by requiring the presence of a significant peak overlapping the gene’s TSS. Peaks were called using MACS2 (v2.1.0)^101^ with the following parameters: –broad –qvalue 0.5.

#### Zccch8 eCLIP analysis

Zcchc8 eCLIP reads were pre-processed using the dockerized environments and ENCODE eCLIP-seq processing pipeline (v2.2) available at https://github.com/YeoLab/eCLIP and as previously described in Van Nostrand et al.^57^ The following modifications were made: After de-multiplexing and adapter trimming, rRNA reads were removed by mapping to rRNA consensus sequences (Dfam DF0000012, DF0000772, DF0001066). Thereafter, rRNA unmapped reads were sorted and mapped against mm10 using STAR with the following parameters to allow for multi-mapping to transposable elements: –outFilterMultimapNmax 100 –winAnchorMultimapNmax 100 –runThreadN 32. Next, CLAM (v1.2.0)^102^ was used to remove PCR duplicates, realign multi-mapping reads using an expectation-maximization approach, and call eCLIP binding peaks. To calculate eCLIP enrichment across gene biotypes (Figure S4E), counts per genomic element were first estimated using TEcount, and the log2FoldChange between input and Zcchc8 IP samples calculated using DESeq2.

#### Premature termination data analysis

Data from poly(A)-tail sequencing experiments conducted in mESCs was downloaded from GEO, processed as described in the respective studies: GEO:GSE178550 (3P-Seq data from WT and Zcchc8 KO mESCs,^31^, GEO:GSE100536 (2P-Seq data from WT and Exosc3 cKO mESCs^28^), GEO:GSE218125 (PAC-Seq data from siNT and siRRP40-treated mESCs^58^). Metagene plots were generated using deepTools, calculating the frequency of termination sites transcriptome-wide across each dataset and respective conditions.

## Supplementary Material

1

2

## Figures and Tables

**KEY RESOURCES TABLE:** 

REAGENT or RESOURCE	SOURCE	IDENTIFIER

Antibodies		

Anti-Histone H3 (acetyl K27) antibody - ChIP Grade	Abcam	Abcam Cat# ab4729, RRID:AB_2118291
Anti-Histone H3 (tri methyl K9) antibody - ChIP Grade	Abcam	ab8898; RRID:AB_306848
Purified anti-RNA Polymerase II RPB1 Antibody (8WG16)	Biolegend	664906; RRID:AB_2565554
Purified anti-RNA Polymerase II RPB1 Antibody (CTD4H8)	Biolegend	904001; RRID:AB_2565036
Anti-Exosc3	Abcam	ab156683; RRID:AB_2619635
anti-Rabbit-HRP	CST	Cat#: 7074S; RRID:AB_2099233
anti-Flag-M2-HRP	Sigma	A8592-.2MG; RRID:AB_439702
anti-B-Actin(8H10D10)-HRP	CST	12262S;RRID: AB_2566811
anti-Zcchc8	Proteintech	Cat #: 23374–1-AP, Lot: 00076174 RRID:AB_2879269
Rabbit IgG	Abcam	Cat #: ab37415, Lot: GR3327091–1 RRID:AB_2631996
Anti-Ints11 antibody	Sigma	#HPA029025; RRID: AB_10600425
Anti-Cpsf2 antibody	Santa Cruz	sc-165983 RRID:AB_2084371
Anti-Rbm7 antibody	Invitrogen	PA5–110280; RRID: AB_2855691

Chemicals, peptides, and recombinant proteins		

DMEM/F12 with Hepes	Gibco	11330032
Neurobasal	Gibco	21103049
N2	Gibco	17502058
Serum-free B27	Gibco	17504044
L-Glutamine	Gibco	25030081
Antibiotic-antimycotic	Gibco	15240062
Bovine Albumin Fraction V	Gibco	15260037
2-Mercaptoethanol	Gibco	21985023
CHIR99021	Reprocell	04–0004
PD03255901	Reprocell	04–0006
Mouse Recombinant LIF	R&D Systems	8878-LF-500/CF
Accutase	Corning	25–058-CI
Human Plasma Fibronectin	EMD Millipore	FC-010–5mg
KnockOut Serum Replacement	Gibco	10828028
Activin A	R&D Systems	338-AC-050/CF
Fgf2	R&D Systems	233-FB-025
4-Hydroxytamoxifen	EMD Millipore	508225
KnockOut DMEM	Gibco	10829018
Sodium Pyruvate	Gibco	11360070
MEM Non-Essential Amino Acids	Gibco	11140050
TRIzol Reagent	Invitrogen	15596026
UltraPure DNase/RNase-Free Distilled Water	Invitrogen	10977015
Halt protease and phosphatase inhibitors	Thermo Scientific	78446
Protein A Dynabeads	Thermo Fisher	10001D
RNase	Invitrogen	AM2271
Proteinase K	Invitrogen	AM2546
Dynabeads MyOne Streptavidin T1	Invitrogen	65601
Murine Rnase Inhibitor	NEB	M0214L
Dynabeads MyOne Silane	Thermo Fisher Scientific	37002D
Turbo DNase 2U/ul	Thermo Fisher Scientific	AM2239
RNaseI 100U/ul	Thermo Fisher Scientific	AM2295
FastAP 1U/ul	Thermo Fisher Scientific	EF0652
T4 PNK 10U/ul	NEB	M0201L
T4 RNA Ligase 1 high conc. 30U/ul	NEB	M0437M
Proteinase K 0.8U/ul	NEB	P8107S
Q5 PCR Master Mix	NEB	M0494L
AffinityScript reverse transcriptase	Agilent	600107
Exo-SAP-IT	Thermo Fisher Scientific	78201
SUPERase⋅ In^™^ RNase Inhibitor	Thermo Fisher Scientific	AM2694
Lipofectamine 2000	Invitrogen	11668030
Lenti-X concentrator	Takara	631231
dTAG-7	Tocris Bioscience	6912

Critical commercial assays		

Direct-zol RNA Miniprep Plus Kit	Zymo Research	R2072
High-Capacity cDNA Reverse Transcription	Applied Biosystems	4368814
SuperScript VILO cDNA synthesis kit	Invitrogen	11754–050
Illumina Stranded Total RNA Prep Kit with Ribo-Zero Plus	Illumina	20020596
MinElute PCR Purification Kit	Qiagen	28004
NEBNext^®^ Ultra II DNA Library Prep Kit	NEB	E7645S
ArimaHiC+ kit	Arima	A101020
Click-iT Nascent RNA Capture Kit	Life Technologies	C10365
PureLink Genomic DNA Mini Kit	Invitrogen	K1820–00
NuPage4–12% BT Gel 1.5mm 10w 10 Per Box	Invitrogen	NP0335BOX
NuPAGE^®^ MOPS SDS Running Buffer (20X)	Invitrogen	NP0001
RNA Clean & Concentrator^™^-5	Zymo Research	R1015
NEBNext^®^ Ultra^™^ II Directional RNA Library Prep Kit	NEB	E7760S
NEBNext^®^ rRNA Depletion Kit (Human/Mouse/Rat)	NEB	E6310L
NEBNext^®^ rRNA Depletion Kit v2 (Human/Mouse/Rat)	NEB	E7400L

Deposited data		

Raw and analyzed data	This paper	GEO:GSE205211
MERVL+/Zscan4+ mESC RNA-Seq	Eckersley-Maslin et al.^36^	GEO:GSE75751
MERVL activation mESC RNA-Seq	Yang et al.^34^	GEO:GSE119819
DUX OE mESC RNA-Seq	Hendrickson et al.^41^	GEO:GSE85627
LSD1 GT mESC RNA-Seq	Agarwal et al.^40^	GEO:GSE94556
miR34 KO mESC RNA-Seq	Choi et al.^39^	GEO:GSE69484
Rbm7 KD mESC RNA-Seq, Rrp40 KD mESC PRO-Seq and CAGE-Seq	Lloret-Llinares et al.^32^	GEO:GSE115727
Zcchc8 KO and Zfc3h1 KO mESC RNA-Seq, MPP8 mESC ChIP-Seq, Zcchc8 KO mESC 3P-Seq	Garland et al.^31^	GEO:GSE178550
Ints11 KD mESC RNA-Seq	Stein et al.^52^	GEO:GSE200702
Mouse embryo RNA-Seq	Qiao et al.^56^	GEO:GSE138760
Exosc3 cKO mESC 2P-Seq	Chiu et al.^28^	GEO:GSE100536
siRrrp40 mESC PAC-Seq	Mimoso and Adelman^58^	GEO:GSE218125

Experimental models: Cell lines		

Exosc3 COIN/COIN mESCs	Gift from Uttiya Basu lab	PMID: 25119026
HEK293T	ATCC	CRL-3216 RRID:CVCL_0063

Oligonucleotides		

ON-TARGETplus Mouse Exosc3 siRNA	Dharmacon Horizon Discovery	L-064537-01-0005
ON-TARGETplus Mouse Zcchc8 siRNA	Dharmacon Horizon Discovery	L-057599-01-0005
ON-TARGETplus Mouse Ints11 siRNA	Dharmacon Horizon Discovery	L-062233-01-0005
ON-TARGETplus Mouse Rbm7 siRNA	Dharmacon Horizon Discovery	L-055957-01-0020
ON-TARGETplus Mouse Dux siRNA	Dharmacon Horizon Discovery	L-161776-00-0010
ON-TARGETplus Mouse Cpsf2 siRNA	Dharmacon Horizon Discovery	L-059334-01-0005

Recombinant DNA		

pLVX-puro_mmExosc3-FKBP	This study	This study
psPAX2	Addgene	Addgene plasmid # 12260; http://n2t.net/addgene:12260; RRID:Addgene_12260
pMD2.G	Addgene	Addgene plasmid # 12259; http://n2t.net/addgene:12259; RRID:Addgene_12259

Software and algorithms		

R (v4.0.3)	https://www.r-project.org/	N/A
Python (v3.8.2)	https://www.python.org/	N/A
Trim Galore (v0.5.0)	Martin	N/A
STAR (v2.7.5b)	Dobin et al.^88^	N/A
TEcount (v2.2.1)	Jin et al.^89^	N/A
DESeq2 (v.1.30.0)	Love et al.^90^	N/A
fgsea (v1.16.0)	Korotkevich et al.^91^	N/A
CellRanger (v5.0.1)	10X Genomics, Inc	N/A
scTE (v1.0.0)	He et al.^92^	N/A
Seurat (v4.0.1)	Stuart et al.^93^	N/A
StringTie (v2.2.0)	Shumate et al.^94^	N/A
Mfuzz (v2.50.0)	Kumar and Futschik^95^	N/A
RepeatMasker (v4.1.1)	https://www.repeatmasker.org/	N/A
MacVector (v18.1)	https://macvector.com/	N/A
bowtie2 (v2.4.1)	Langmead and Salzberg^96^	N/A
sambamba (v0.5.6)	Tarasov et al.^97^	N/A
deepTools (v3.5.0)	Ramirez et al.^98^	N/A
seqkit (v0.10.1)	Shen et al.^99^	N/A
velociraptor (v1.0.0)	https://github.com/kevinrue/velociraptor	N/A
GenoSTAN (v2.18.0)	Zacher et al.^100^	N/A
MACS2 (v2.1.0)	Zhang et al.^101^	N/A
ENCODE eCLIP-seq processing pipeline (v2.2)	Van Nostrand et al.^57^	N/A
CLAM (v1.2.0)	Zhang and Xing^102^	N/A
HiC-Pro	Servant et al.^103^	N/A
HOMER	Heinz et al.^104^	N/A
FitHiChIP	Bhattacharyya et al.^105^	N/A
hicExplorer	Ramirez et al.^98^	N/A

Other		

micro AFA Fiber Crimp-Cap 6x16mm tubes	Covaris	520091
Ampure XP beads	Beckman Coulter	A63881

## Highlights

- The nRE and INT maintain ES identity
- Loss of nRE and INT induce a 2C-like totipotent cell state
- nRE and INT suppress ERV expression
- nRE and INT suppress non-productive transcription
