# Supplementary material for: Nuclear RNA catabolism controls endogenous retroviruses, gene expression asymmetry, and dedifferentiation
Source: Mol Cell. Author manuscript; Available in PMC 2024 Feb 5. (PMC10842741; doi:10.1016/j.molcel.2023.10.036)
Supplement: 1 [file NIHMS1950659-supplement-1.pdf]

Molecular Cell, Volume *83*

Supplemental information

Nuclear RNA catabolism controls endogenous retroviruses, gene expression asymmetry, and dedifferentiation

Denis Torre, Yesai S. Fstkchyan, Jessica Sook Yuin Ho, Youngseo Cheon, Roosheel S. Patel, Emma J. Degrace, Slim Mzoughi, Megan Schwarz, Kevin Mohammed, Ji-Seon Seo, Raquel Romero-Bueno, Deniz Demircioglu, Dan Hasson, Weijing Tang, Sameehan U. Mahajani, Laura Campisi, Simin Zheng, Won-Suk Song, Ying-chih Wang, Hardik Shah, Nancy Francoeur, Juan Soto, Zelda Salfati, Matthew T. Weirauch, Peter Warburton, Kristin Beaumont, Melissa L. Smith, Lubbertus Mulder, S. Armando Villalta, Kai Kessenbrock, Cholsoon Jang, Daeyoup Lee, Silvia De Rubeis, Inma Cobos, Oliver Tam, Molly Gale Hammell, Marcus Seldin, Yongsheng Shi, Uttiya Basu, Vittorio Sebastiano, Minji Byun, Robert Sebra, Brad R. Rosenberg, Chris Benner, Ernesto Guccione, and Ivan Marazzi

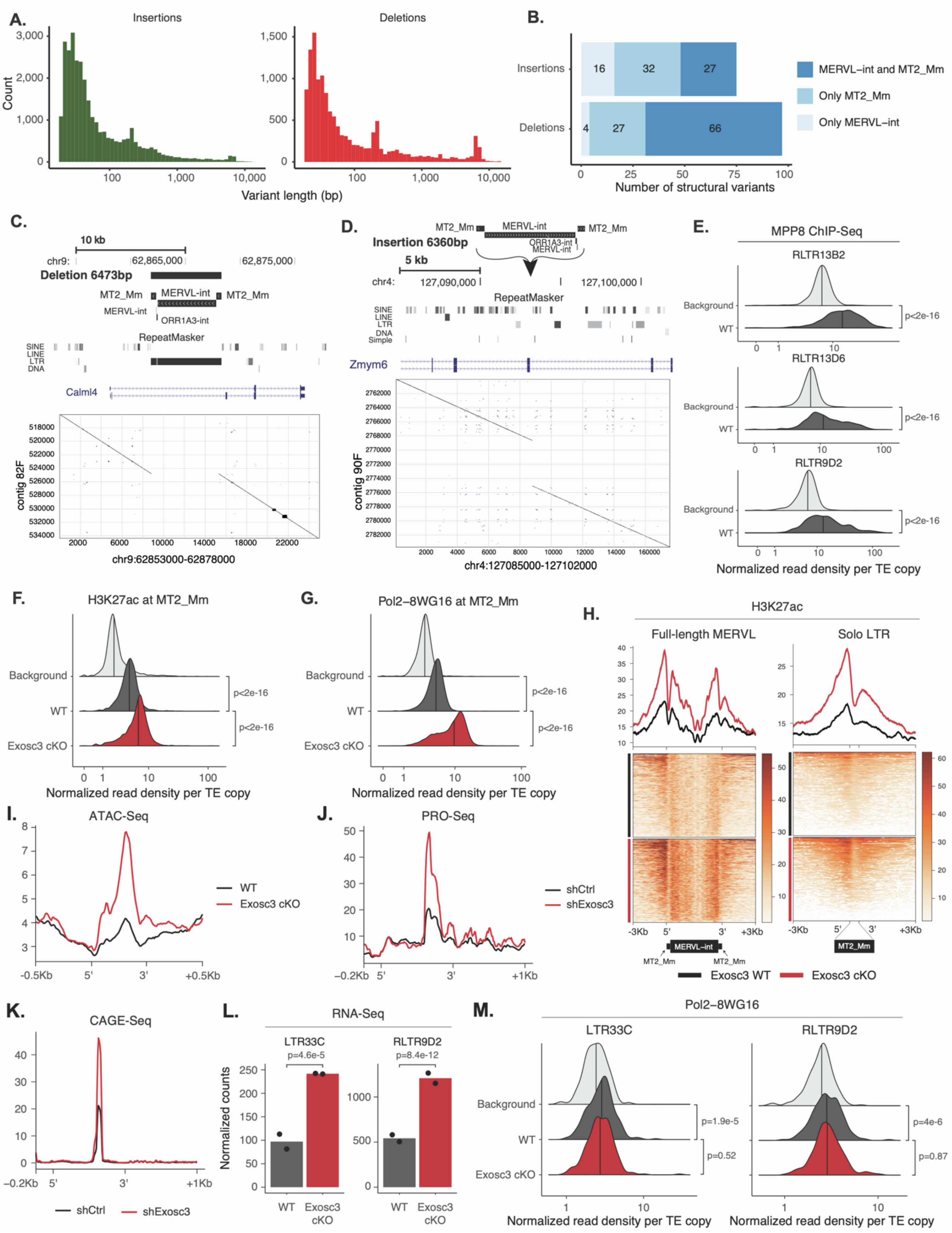

A.
Insertions
Deletions
Count
Variant length (bp)
B.
Insertions
Deletions
MERVL–int and MT2_Mm
Only MT2_Mm
Only MERVL–int
Number of structural variants
C.
10 kb
chr9:
62,865,000
62,875,000
Deletion 6473bp
MT2_Mm
MERVL-int
MT2_Mm
MERVL-int
ORR1A3-int
RepeatMasker
SINE
LINE
LTR
DNA
Calml4
contig 82F
chr9:62853000-62878000
D.
MT2_Mm
MERVL-int
MT2_Mm
Insertion 6360bp
ORR1A3-int
MERVL-int
5 kb
chr4:
127,090,000
127,100,000
RepeatMasker
SINE
LINE
LTR
DNA
Simple
Zmym6
contig 90F
chr4:127085000-127102000
E.
MPP8 ChIP-Seq
RLTR13B2
RLTR13D6
RLTR9D2
Background
WT
p<2e-16
Normalized read density per TE copy
F.
H3K27ac at MT2_Mm
Background
WT
Exosc3 cKO
p<2e-16
p<2e-16
Normalized read density per TE copy
G.
Pol2–8WG16 at MT2_Mm
Background
WT
Exosc3 cKO
p<2e-16
p<2e-16
Normalized read density per TE copy
H.
H3K27ac
Full-length MERVL
Solo LTR
-3Kb
5'
3'
+3Kb
MERVL–int
MT2_Mm
MT2_Mm
MT2_Mm
Exosc3 WT
Exosc3 cKO
I.
ATAC-Seq
WT
Exosc3 cKO
-0.5Kb
5'
3'
+0.5Kb
J.
PRO-Seq
shCtrl
shExosc3
–0.2Kb
5'
3'
+1Kb
K.
CAGE-Seq
–0.2Kb
5'
3'
+1Kb
shCtrl
shExosc3
L.
RNA-Seq
LTR33C
p=4.6e-5
RLTR9D2
p=8.4e-12
Normalized counts
WT
Exosc3 cKO
M.
Pol2–8WG16
LTR33C
RLTR9D2
Background
WT
Exosc3 cKO
p=1.9e-5
p=0.52
p=4e-6
p=0.87
Normalized read density per TE copy

Figure S1 – Multi-omic characterization of TEs in WT and *Exosc3* cKO mESCs, related to Figure 1

A. Distribution of structural variant lengths in the PacBio de novo sequenced cognate genome of *Exosc3* COIN mESCs compared to mm10
B. Number of newly inserted and deleted copies of full-length MERVL, solo MT2_Mm, or solo MERVL-int fragments in PacBio de novo sequenced cognate genome of *Exosc3* COIN mESC's compared to mm10
C. and D. Examples of full-length MERVL deletion and insertion events in the PacBio de novo sequenced cognate genome of *Exosc3* COIN mESCs compared to mm10
E. Density plots displaying normalized MPP8 ChIP-Seq reads (from Garland et al.) across genomic copies of RLTR13B2, RLTR13D6, RLTR9D2 in WT mESCs compared to background (see Methods for details). P-values were calculated using an unpaired, two-sided Wilcoxon rank-sum test and adjusted using the Benjamini-Hochberg method
F. and G. Distribution H3K27ac and RNAPII (8WG16 antibody) ChIP-Seq signal at MT2_Mm loci, in WT and *Exosc3* cKO cells, compared to background
H. Enrichment of H3K27ac ChIP-Seq pileups at full-length MERVL elements (MERVL-int flanked by two MT2_Mm LTRs) and solo LTRs (MT2_Mm not located in proximity of full-length MERVLs), in control and *Exosc3* cKO mESCs
I. Enrichment of ATAC-Seq pileups at full-length MERVL and solo MT2_Mm elements in WT and *Exosc3* cKO mESCs
J. and K. As above, but showing PRO-Seq and CAGE-seq data in siCtrl and siExosc3 data (from Lloret-Llinares et al.)
L. Distribution of RNAPII ChIP-Seq signal at LTR33C and LTR9D2 TE copies, in WT and *Exosc3* cKO cells, compared to background. p-values were calculated using DESeq2 and adjusted using the Benjamini-Hochberg method.
M. Enrichment of RNAPII ChIP-Seq pileups at LTR33C and LTR9D2 TE copies, in WT and *Exosc3* cKO mESCs

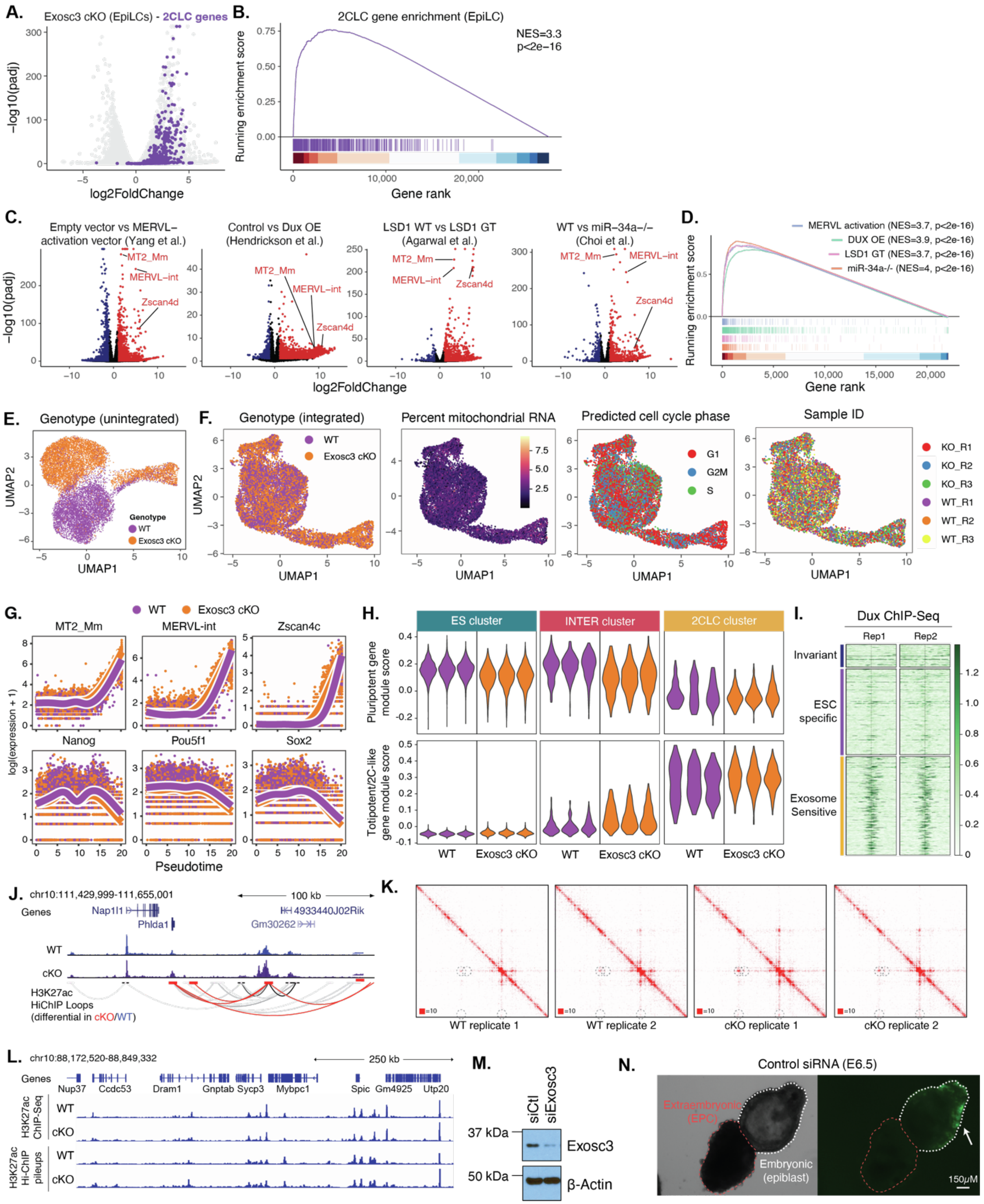

Figure S2 – *Exosc3* loss promotes a 2CLC phenotype, related to Figure 2

A. Volcano plot of differentially expressed genes and TEs between WT and *Exosc3* cKO EpiLCs. 2CLC-specific genes and TEs, representing markers of 2CLCs, are highlighted in purple (defined from Eckersley-Maslin et al. 2016). Other genes and TEs are marked in grey

B. Gene set enrichment analysis (GSEA) of 2CLC genes/TEs in *Exosc3* cKO EpiLCs. Genes/TEs are ranked according to the differential expression statistic (DESeq2 Wald test, lower rank – higher expression in cKO, higher rank – lower expression in cKO). Color bar displays differential expression statistic values. (NES – normalized enrichment score).
C. Volcano plots of showing differentially expressed genes and TEs (|log2-fold change| > 1, p-value < 0.05) across perturbations known to induce a 2CLC state in multiple published datasets
D. GSEA analysis of upregulated gene/TE sets identified from **Figure S2C** in the differential expression signature between WT vs *Exosc3* cKO mESCs
E. UMAP (unintegrated data) of mESCs, with points colored by genotype
F. UMAP (integrated data) of mESCs, with points colored by genotype, sample ID and selected QC metrics, demonstrating consistent data integration
G. Normalized expression of selected genes and TEs over single-cell pseudotime, colored by genotype
H. Profile of ES, INTER and 2CLC based on genotype and pluri- vs totipotent gene modules. ES: embryonic stem cell (blue); INTER: Intermediate state, (red); 2CLC: 2C like cells, (yellow). Purple: WT, orange: *Exosc3* cKO
I. Heatmap displaying Dux4 ChIP-Seq pileups in mESCs at enhancer clusters from **Figure 2G**
J. Genome browser snapshot of HiChIP loops stronger in WT and *Exosc3* cKO mESCs
K. Heatmap displaying Hi-ChIP contact matrix across replicates for genomic region in **Figure S2J**
L. Genome browser snapshot displaying concordance between H3K27ac ChIP-Seq and H3K27ac Hi-ChIP signal
M. Western blot displaying depletion of Exosc3 protein in siCtrl and siExosc3 mESCs
N. Embryo injected with control siRNAs transfected cells showing contribution to embryonic (epiblast) but not extraembryonic (EPC – ectoplacental cone) compartments

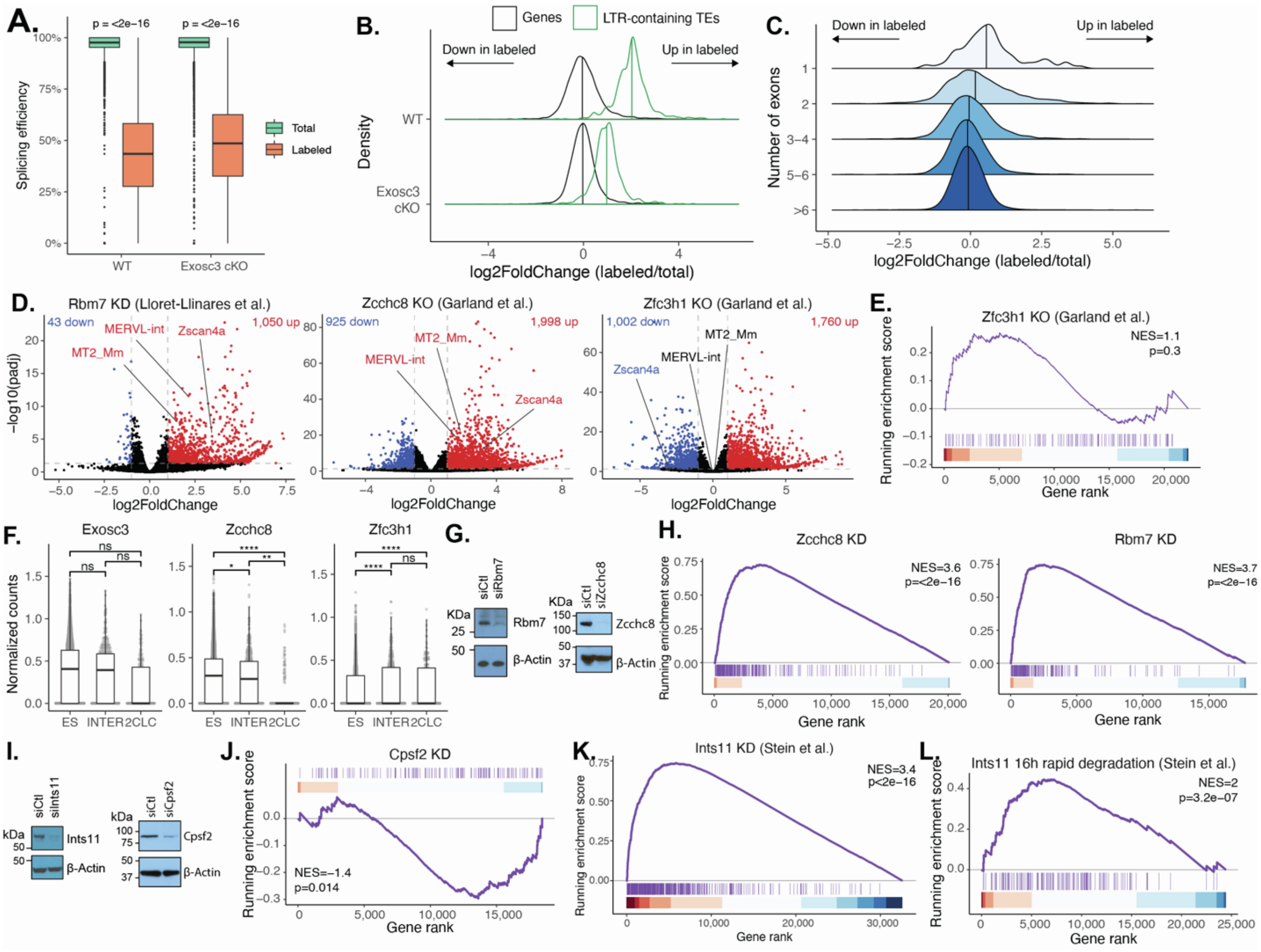

Figure S3 – Transcriptomic changes upon loss of RNA catabolism and cleavage complexes, related to Figure 3

A. Box plots displaying splicing efficiency in the metabolically labeled (TT-Seq) and unlabeled RNA fractions for control WT and *Exosc3* cKO mESCs
B. Density plots displaying the distribution of log2-fold changes from a differential expression analysis of EU-labeled versus unlabeled RNA in WT and *Exosc3* cKO mESC. Positive fold changes indicate higher expression in labeled RNA
C. Density plots displaying the distribution of log2-fold changes of genes from a differential expression analysis of EU-labeled versus unlabeled RNA in WT and *Exosc3* cKO mESCs, grouped by number of exons. Positive fold changes indicate higher expression in labeled RNA
D. Volcano plot displaying differentially expressed genes and TEs in *Rbm7* KD, *Zcchc8* KO, and *Zfc3h1* KO mESCs (from Garland et al., Lloret-Llinares et al.)
E. GSEA analysis of 2CLC genes/TEs for the differential expression signature of *Zfc3h1* KO mESCs (from **Figure S3D**)
F. Box plots displaying normalized expression of *Exosc3, Zcchc8* and *Zfc3h1* in cells from ES, INTER and 2CLC clusters from 10X scRNA-Seq data (cluster assignments from **Figure 2C**). Reported p-values were calculated using DESeq2 from pseudobulk data and adjusted using the Benjamini-Hochberg method
G. Western blots confirming Zcchc8 and Rbm7 KD by siRNA in mESCs
H. GSEA analysis of 2CLC genes/TEs in differential expression signatures from *Zcchc8* KD and *Rbm7* KD mESCs
I. Western blots confirming Ints11 and Cpsf2 KD by siRNA in mESCs
J. GSEA analysis of 2CLC genes/TEs in differential expression signatures from *Cpsf2* KD mESCs

K. GSEA analysis of 2CLC genes/TEs in differential expression signatures from *Ints11* KD mESCs (48h knock-down, data from Stein et al.)
L. GSEA analysis of 2CLC genes *Ints11* rapid depletion mESCs (16h depletion, Stein et al.)

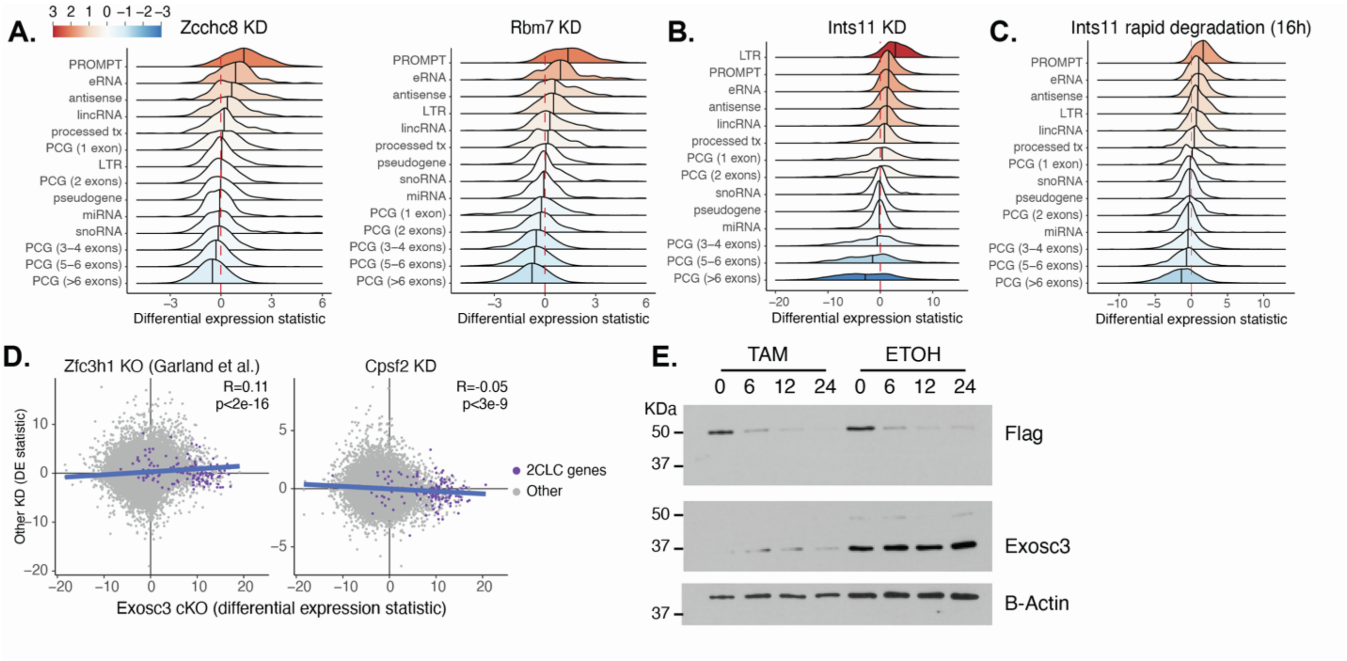

Figure S4 – Gene asymmetry upon loss of NEXT and Integrator complexes, related to Figure 4

A. Density plots displaying the differential expression statistic (Wald test, from DESeq2) from the *Zcchc8* KD and *Rbm7* KD differential gene and TE expression signatures in **Figures S3H**, grouped by gene biotype. Protein-coding genes are further grouped by the number of exons.
B. As above, but for the *Ints11* KD signature described in **Figure S3K**
C. As above, but for the Ints11 rapid degradation signature described in **Figure S3L**
D. Scatter plot displaying correlation between the gene/TE differential expression signatures of *Exosc3* cKO (x axis) and *Zfc3h1* KO and *Cpsf2* KD (y axis, left and right respectively) in mESCs
E. Western blot displaying depletion of Exosc3 from the rapid degradation experiment

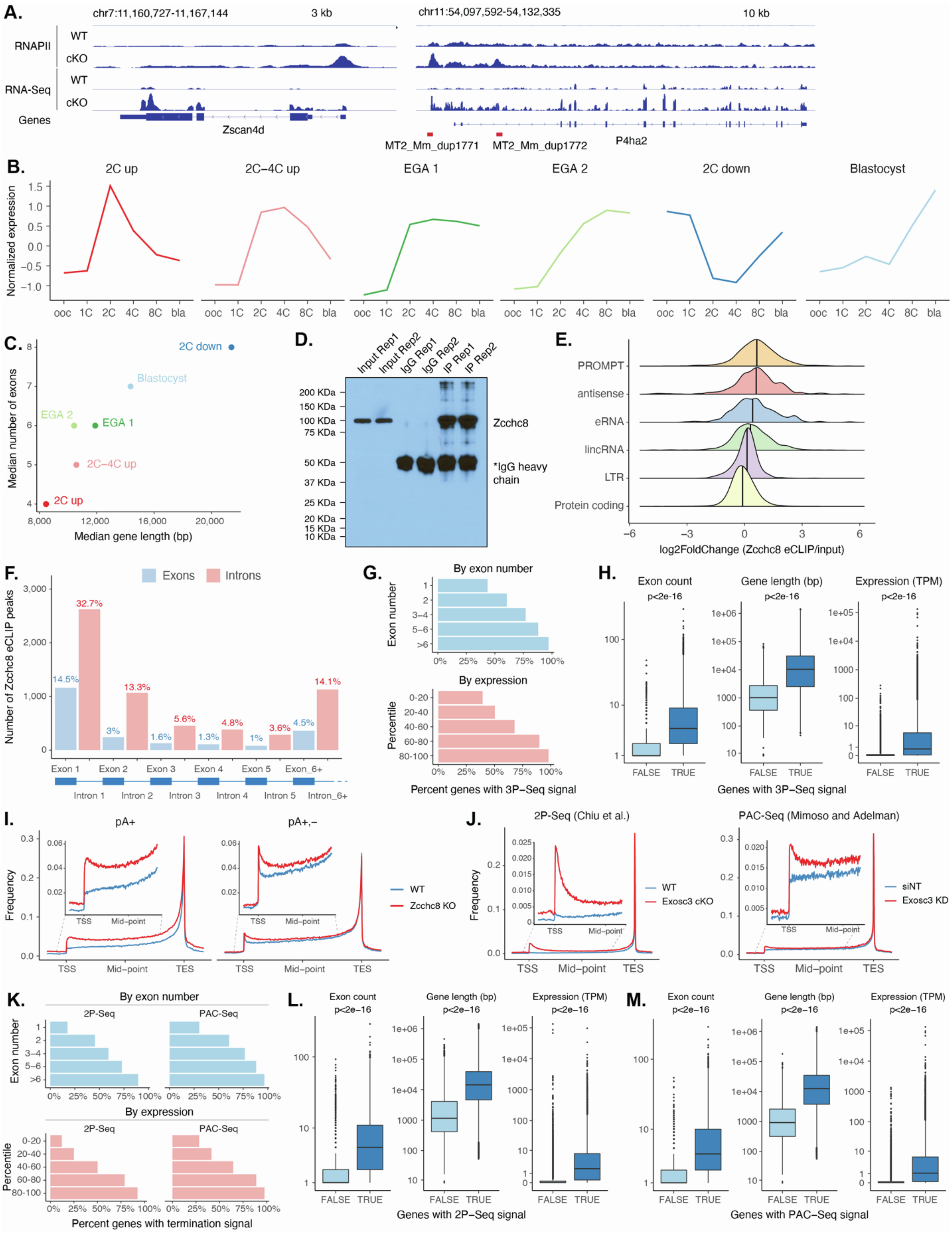

A.
chr7:11,160,727-11,167,144
3 kb
chr11:54,097,592-54,132,335
10 kb
RNAPII
WT
cKO
RNA-Seq
WT
cKO
Genes
Zscan4d
MT2_Mm_dup1771
MT2_Mm_dup1772
P4ha2
B.
2C up
2C−4C up
EGA 1
EGA 2
2C down
Blastocyst
Normalized expression
ooc 1C 2C 4C 8C bla
C.
2C down
Blastocyst
EGA 2
EGA 1
2C−4C up
2C up
Median number of exons
Median gene length (bp)
D.
Input Rep1
Input Rep2
IgG Rep1
IgG Rep2
IP Rep1
IP Rep2
200 KDa
150 KDa
100 KDa
75 KDa
50 KDa
37 KDa
25 KDa
20 KDa
15 KDa
10 KDa
Zcchc8
*IgG heavy chain
E.
PROMPT
antisense
eRNA
lincRNA
LTR
Protein coding
log2FoldChange (Zcchc8 eCLIP/input)
F.
Exons
Introns
Number of Zcchc8 eCLIP peaks
14.5%
32.7%
3%
13.3%
1.6%
5.6%
1.3%
4.8%
1%
3.6%
4.5%
14.1%
Exon 1
Exon 2
Exon 3
Exon 4
Exon 5
Exon_6+
Intron 1
Intron 2
Intron 3
Intron 4
Intron 5
Intron_6+
G.
By exon number
Exon number
By expression
Percentile
Percent genes with 3P−Seq signal
H.
Exon count
Gene length (bp)
Expression (TPM)
p<2e-16
FALSE
TRUE
Genes with 3P−Seq signal
I.
pA+
pA+,−
Frequency
TSS
Mid−point
TES
WT
Zcchc8 KO
J.
2P-Seq (Chiu et al.)
PAC-Seq (Mimoso and Adelman)
WT
Exosc3 cKO
siNT
Exosc3 KD
K.
By exon number
2P-Seq
PAC-Seq
Exon number
By expression
Percentile
Percent genes with termination signal
L.
Exon count
Gene length (bp)
Expression (TPM)
p<2e−16
Genes with 2P−Seq signal
M.
Exon count
Gene length (bp)
Expression (TPM)
p<2e−16
Genes with PAC−Seq signal

Figure S5 – RNA exosome and NEXT complex regulate premature termination, related to Figure 5

A. Genome browser snapshot of displaying long, multi-exonic protein-coding genes with higher levels of RNAPII (8WG16 antibody) and RNA-Seq upregulation in Exosc3 cKO mESCs, when compared to WT
B. Line plots displaying normalized expression of clusters of genes detected in mouse embryo RNA-Seq data (data from Qiao et al., see Methods for details)
C. Scatter plot displaying median gene length and exon counts for gene clusters in (**Figure S4B**)
D. Immunoprecipitation-western blot validation of anti-Zcchc8 antibody pull-down efficiency in UV-crosslinked COIN cell lysates
E. Density plot displaying the log2-fold changes (input vs eCLIP) of Zcchc8 binding across gene biotypes
F. Bar plot displaying the percentage of Zcchc8 eCLIP peaks located within exons and introns at increasing positions with respect to the TSS, across the transcriptome
G. Bar plots displaying the percentage of genes with detected 3P-Seq signal (WT mESCs, pA+,- fraction), grouped by number of exons (top) or by expression level (bottom)
H. Box plots displaying the distribution of exon number (left), total gene length (middle), and average expression in Transcripts Per Million (TPM, right) of genes with and without detected 3P-Seq signal (WT mESCs, pA+,- fraction). P-values calculated using an unpaired, two-sided Wilcoxon test and adjusted using the Benjamini-Hochberg method.
I. Metagene plot displaying the frequency of unique pA+ and pA+,- termination sites in WT and *Zcchc8* KO mESCs
J. Metagene plot displaying the frequency of unique pA+ termination sites in WT and *Exosc3* KO mESCs (2P-Seq, data from Chiu et al., left); and control and *Exosc3* KD mESCs (PAC-Seq, data from Mimoso & Adelman, right)
K. Bar plots displaying the percentage of genes with detected 2P-Seq signal or PAC-Seq signal (data from **Figure S4J**), grouped by number of exons (top) or by expression level (bottom)
L. Box plots displaying the distribution of exon number (left), total gene length (middle), and average expression in Transcripts Per Million (TPM, right) of genes with and without detected 2P-Seq signal. P-values calculated using an unpaired, two-sided Wilcoxon test and adjusted using the Benjamini-Hochberg method.
M. As above, but using data from PAC-Seq
